# Soil-derived bacteria endow *Camellia* weevil with more ability to resist plant chemical defense

**DOI:** 10.1186/s40168-022-01290-3

**Published:** 2022-06-25

**Authors:** Shouke Zhang, Zikun Li, Jinping Shu, Huaijun Xue, Kai Guo, Xudong Zhou

**Affiliations:** 1 State Key Laboratory of Subtropical Silviculture, Zhejiang A&F University, Zhejiang, Hangzhou 311300 People’s Republic of China; 2 College of Forestry and Biotechnology, Zhejiang A&F University, Zhejiang, Hangzhou 311300 People’s Republic of China; 3 Research Institute of Subtropical Forestry, Chinese Academy of Forestry, Zhejiang, Hangzhou 311400 People’s Republic of China; 4 College of Life Sciences, Nankai University, Tianjin, 300071 People’s Republic of China

**Keywords:** *Acinetobacter*, *Curculio chinensis*, Gut microbial communities, Soil microorganisms, Plant secondary metabolite degradation, Tea saponin

## Abstract

**Background:**

Herbivorous insects acquire their gut microbiota from diverse sources, and these microorganisms play significant roles in insect hosts’ tolerance to plant secondary defensive compounds. *Camellia* weevil (*Curculio chinensis*) (CW) is an obligate seed parasite of *Camellia oleifera* plants. Our previous study linked the CW’s gut microbiome to the tolerance of the tea saponin (TS) in *C. oleifera* seeds. However, the source of these gut microbiomes, the key bacteria involved in TS tolerance, and the degradation functions of these bacteria remain unresolved.

**Results:**

Our study indicated that CW gut microbiome was more affected by the microbiome from soil than that from fruits. The soil-derived *Acinetobacter* served as the core bacterial genus, and *Acinetobacter* sp. was putatively regarded responsible for the saponin-degradation in CW guts. Subsequent experiments using fluorescently labeled cultures verified that the isolate *Acinetobacter* sp. AS23 can migrate into CW larval guts, and ultimately endow its host with the ability to degrade saponin, thereby allowing CW to subsist as a pest within plant fruits resisting to higher concentration of defensive chemical.

**Conclusions:**

The systematic studies of the sources of gut microorganisms, the screening of taxa involved in plant secondary metabolite degradation, and the investigation of bacteria responsible for CW toxicity mitigation provide clarified evidence that the intestinal microorganisms can mediate the tolerance of herbivorous insects against plant toxins.

Video Abstract

**Supplementary Information:**

The online version contains supplementary material available at 10.1186/s40168-022-01290-3.

## Background

Plants have evolved into various physical and chemical phenotypes to resist insect damages during the process of their coevolution [1–3]. Phytochemical resistance mechanisms involve toxic, anti-nutritional, and indigestion-promoting compounds produced by plants that are deployed in response to the feeding of herbivorous insects, or that steadily accumulated over a long period of time [3]. These compounds generally inhibit pest activity, affect pest growth, or perturb their digestive systems [4–6], ultimately playing important roles in anti-insect defenses [7, 8]. In addition to the roles of digestive and detoxification enzymes in the digestive tract of herbivore insects [3, 9–11], insect gut microbiota is the important third interacting party in detoxification of phytochemical defensive compounds and must be considered when evaluating insect resistance to plant secondary metabolites [2, 7, 12–14]. Microorganisms in insect guts can promote hosts to digest nutrients and facilitate phytophagous insects’ adaptation to the plant secondary metabolites quickly [4, 15–18] by helping insect hosts effectively degrade or avoid the toxic chemicals produced by host plants [2, 9, 19–22]. However, difficulties in investigating the host-microbiome-plant systems and inadequate methodological capabilities have limited the research into the microbial taxa that may have functional roles in such interactions [23–25].

Microbiomes in herbivorous insect guts are influenced by environments, insect diets, and their unique feeding characteristics [26]. Complex sources of microbiomes further hinder the investigations into the symbioses of gut microbiomes of herbivorous insects in the adaptation to plant hosts [26]. The gut microorganisms of herbivorous insects can metabolize plant toxins and clearly perform critical roles, especially for the microbes recruited naturally from both plant and soil [27]. It is a labile trait that soil microorganisms metabolize the plant toxins, and the microorganisms can spread inside plant leaves, ultimately into the guts of herbivorous insects [27]. Moreover, further studies confirmed that some microbiota strains can produce unique hydrolytic enzymes which help insect degrade plant toxic compounds in its gut systems [4, 28]. Except for few studies concerning the role of bacterial strains on insect host adaptation [29, 30], most studies solely used molecular methods including 16S rRNA gene high-throughput sequencing (HTS) and metagenomics to obtain data and speculatively described the interactions and the species of the putative functional microbiome [31, 32]. Consequently, lack of functional verification test of the microbiome activities has led to uncertain and speculative conclusions without direct evidence [31, 32].

**Fig. 1:**
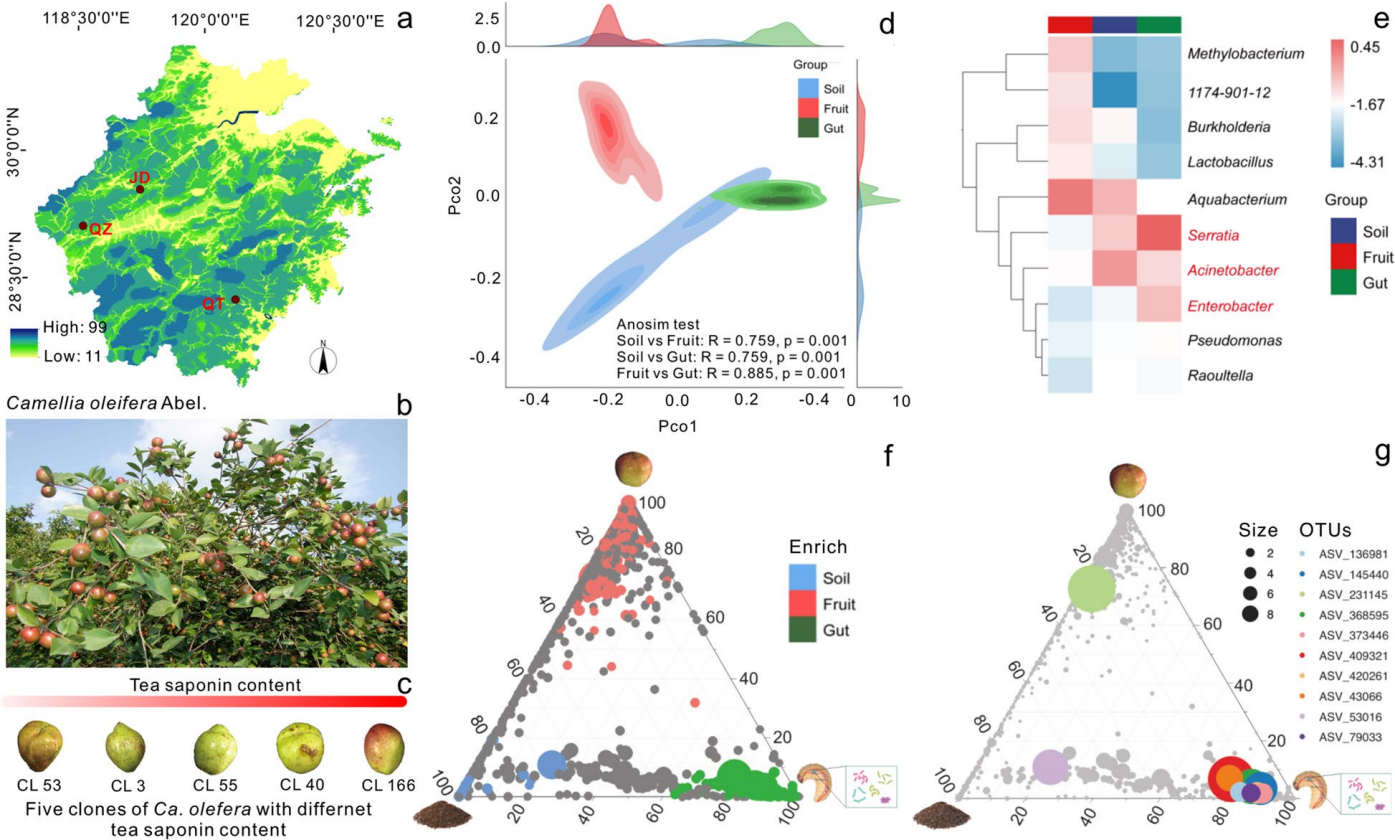
Sample collection information and microbiome diversity and species composition analysis from fruit, soil, and weevil gut microbiomes. **a** Geographic distribution of sampling locations. Three plantations (QT: Qiangtian, QZ: Quzhou, and JD: Jiande) chosen for experiment. **b** Images of *C. oleifera*. **c** Images of *C. oleifera* clones’ fruits with saponin content increase. Five *C. oleifera* clones (CL 3: Changlin 3, CL 40: Changlin 40, CL 53: Changlin 53, CL 55: Changlin 55, and CL 166: Changlin 166) selected for the study. **d** Unconstrained PCoA ordination based on Bray–Curtis distances showing that three community types are completely disparate from each other (*p* = 0.001, *PERMANOVA* test and *Anosim* test). **e** Top 10 of the most abundant discriminatory bacterial genera were identified by applying random-forest classification of ASV relative abundances among sample types. Biomarker taxa are ranked in descending order of importance based on the classification model. **f** Ternary plots of amplicon sequence variants (ASVs) identified in all samples and their relative abundances. Each point represents a single ASV. Only ASVs found in at least 10% of the samples are included in the figure. The size of each symbol indicates the relative abundance (weighted average) of the ASVs, and its color indicates the sample type. Green depicts ASVs found in > 50% in gut samples, red depicts ASVs found in > 50% in fruit samples, blue depicts ASVs found in > 50% of soil samples, and grey symbols indicate general ASVs found in all sample types. The position of each point reflects its contribution to the total relative abundances of the indicated sample types. **g** Top 10 of the most abundant ASVs are re-colored from fruit, soil, and weevil gut microbiomes. Only ASVs found in at least 10% of the samples are included in the figure. The size of each symbol indicates the relative abundance (weighted average) of the ASV, and its color indicates top 10 of the most abundant ASVs. The position of each point reflects its contribution to the total relative abundances of the indicated sample types

We previously investigated the interactions between an important woody oil crop (*Camellia oleifera*) in South China and its seed parasite, *Camellia* weevil (*Curculio chinensis*) (CW) (Fig. 1a−c) [21]. CWs are a unique group of beetles (Coleoptera) that exhibit special life cycle (pupation and emergence occur inside the soil), mouthparts, and monophagous feeding characteristics [21]. CW larvae live in completely enclosed tea fruits and are entirely isolated from outside environments. CW larvae possess typical chewing mouthparts, and the gut microbiota of fully-grown larva are more sensitive to host diets than plant endophytic bacteria due to in closed space [21]. Tea saponin (TS) which is rich as a triterpene saponin in *C. oleifera* seeds represents the primary compound involved in chemical defense and resistance to CW feeding [21]. Our previous results indicated that gut bacteria of CW larva could help the pest overcome the phytochemical resistance [21]. However, several key questions remain unanswered, (1) where is the gut microbiome that helps CW larvae mitigate plant toxins derived from? (2) are there any key bacterial populations within CW gut microbiomes related to TS toxicity reducing? (3) can the key bacterial populations degrade TS?

The pupa chamber of CW is made of soil and requires opening via mouthparts during the adult emergence, critically involved with the interaction between the soil microorganisms and CW adult gut microbes. The adults also contact the fruit microbiomes during the process of feeding after their emergence. The larval microbiomes could possibly be obtained from soil and/or fruits through the mother before hatching inside *Camellia* fruit. Thus, we investigated the microbiota from fruits, soil, and newly emerged CW adult gut microbiomes using 16S rRNA gene sequencing methods to identify the source of CW gut microbiomes. We further developed an experimental framework using plant clones (i.e., clones CL3, CL40, CL53, CL55, and CL166 of *C. oleifera*) exhibiting different levels of TS content, to compare the effects of TS content on the gut microbiome structure of CWs using genome-resolved metagenomics. Finally, isolation of the key bacteria that could metabolize plant toxins was conducted along with experimental transplantation into sterile CW guts to assess saponin degradation activities.

## Materials and methods

### Sample collection and processing

Three plantations (Qingtian, Zhejiang, 28°11′51.61′′ N, 120°23′15.25′′ E; Quzhou, Zhejiang, 29°3′48′′ N, 118°36′15′′ E; Jiande, Zhejiang, 29°01′ 32.06′′N, 119°37′ 28.45′′E) over 10 years of *C. oleifera* cultivation without human intervention were chosen for sampling (Fig. 1a). Each location was roughly 15 ha in size and contained five *C. oleifera* clones (CL 3, CL 40, CL 53, CL 55, and CL 166) that were selected for study based on fruit saponin content (Fig. 1b). Samples were collected in early June during the peak of CW adults’ emergence. Soil samples, newly emerged adults, and fruits were collected from each *C. oleifera* clone at the same time. To collect CW adults, *Camellia* and the 4 m^2^ of ground under the trees were covered with plastic nets. The newly emerged adult weevils could thus be blocked within the net and easily be captured when they climbed to *C. oleifera* fruits. Eighteen pairs of adults were collected from each clone and the same number of adults were collected from each location using the same method. From each location, 90 pairs (18 pairs × 5 clones) of adult samples were obtained. A total of 270 adult pairs (18 pairs × 5 clones × 3 locations) were collected. When collecting adult insects, five points were randomly selected under the 4 m^2^ net and soil of 20 cm underground was collected and mixed. The total of six soil samples was collected for each clone, respectively. Thirty soil samples were obtained under five clones from each location and 90 soil samples in total (6 soil samples × 5 clones × 3 locations) were collected. After collecting soil samples, young and tender fruits were randomly collected from selected clonal plants. Six fruit samples were collected from each clone and each sample comprising of 10 fruits for subsequent analyses of microbial diversity and TS content. The same number of samples were collected from each location, and in total 90 fruit samples (6 fruit samples × 5 clones × 3 locations) were obtained. All samples were stored in sterile containers, followed by storing at – 80 °C for further experiments.

### DNA extraction, PCR amplification, and DNA sequencing

Soil, adult gut, and fruit samples were treated according to the methods described in Hannula et al. (2019) [27]. DNA extraction was conducted using the QIAamp Fast DNA Stool Mini Kit (QIAGEN, Germany) following the manufacturer’s instructions. The V3−V4 hypervariable regions of 16S rRNA genes were amplified with the universal primers 341F (5′-CCTAYGGGRBGCASCAG-3′) and 806R (5′-GGACTACNNGGGTATCTAAT-3′)^21^. PCR conditions consisted of 94 °C for 2 min followed by 30 cycles at 98 °C for 10 s, 62 °C for 30 s, and 68 °C for 30 s; followed by a final extension at 68 °C for 5 min. The Ion Plus Fragment Library Kit 48 RXNS Kit (Thermofisher, USA) was used to construct sequencing libraries. Libraries were evaluated with a Qubit instrument and then sequenced on the Illumina Novaseq 6000 PE250 platform. All procedures were performed in a sterile environment. Demultiplexed 16S rRNA gene high throughput sequencing raw data are available in the NCBI Sequence Read Archive (Bio-Project ID: PRJNA777383).

Raw reads were filtered using FASTP (version 0.18.0) [33] to remove reads containing > 10% unknown nucleotides, and those with < 50% of bases exhibiting quality values > 20. Paired-end clean reads were then merged using FLASH (version 1.2.11), specifying a minimum overlap of 10 bp and a mismatch error rate of 2% [34]. Poor quality sequences were removed using the QIIME2 pipeline including (1) identification of low-quality regions (default minimum quality ≤ 3; default minimum length ≥ 3) followed by splitting of reads at the first low-quality base in the region; and (2) removal of reads where the length of the continuous sequence of high-quality bases was < 75% of the entire read length. Chimeric sequences were identified among clean reads by searching against a reference database (version r20110519) using UCHIME [35] and removed. The remaining clean reads were used for further analysis. The quality-filtered clean reads were clustered into operational taxonomic units (ASVs) at a ≥ 97% nucleotide similarity threshold using UPARSE (version 9.2.64) pipeline [35]. The sequence with the highest abundance within each ASV cluster was selected as the representative sequence for the ASV. Representative sequences were taxonomically identified using a naive Bayesian model within the RDP classifier (version 2.2) by reference against the SILVA database (version 132) [36] using the confidence threshold of 0.8 [37].

### Microbiome profiling

To ensure that sequencing depth met the analytical requirement, ASV rarefaction and rank abundance curves were plotted using the ggplot2 R package (version 2.2.1) [38, 39]. Subsequently, stacked bar plots of community composition were visualized using the ggplot2 R package (version 2.2.1) [38]. To determine the taxa with the most discriminatory abundances across soil, adult gut, and fruit communities, the relative abundances of bacterial taxa at the genus level were evaluated using the random-forest package v.4.6–14 of R with default parameters [39]. Ternary plots were constructed using the ggtern package (version 3.1.0) [40] to identify abundant microbial populations shared among the three sample types.

Alpha diversity indices (including Chao1, Faith’s phylogenetic diversity, Good’s coverage, Shannon-Wiener, Simpson, Pielou’s evenness, and observed species) were calculated using QIIME2 to investigate the diversity differences among samples. Differences in alpha diversity among the three sample types were compared using Welch’s *t* test, and Wilcoxon’s rank test in the vegan R package (version 2.5.3) [41]. Bray-Curtis’ dissimilarities were calculated between sample communities using the vegan R package (version 2.5.3) [41] to compare their compositional differences. Multivariate statistical tests (Anosim and PERMANOVA), nonparametric multivariate analysis (Adonis), and principal coordinates analysis (PCoA) were calculated using the vegan R package (version 2.5.3) and the Bray-Curtis [41]. Ordinations were plotted using the ggplot2 R package (version 2.2.1) [41]. Regional differences in microbiome diversity were further investigated among samples taken from the three regions using the above-described methods.

### Comparison of microbiomes among sample communities

Circular layout representations of genera abundances were produced using Circos (version 0.69-3) to compare microbial species compositions of soil and adult weevil gut communities from each tree clone [38]. Circular plots were generated using the dynamic real-time interactive Omicsmart platform for data analysis (http://www.omicsmart.com), followed by visualization with R (version 1.1.2) [38]. Multivariate statistical analyses of Bray-Curtis distances among sample communities associated with different tree clones were conducted and included principal coordinates analysis (PCoA) using the vegan R package (version 2.5.3) [41], followed by visualization with the ggplot2 R package (version 2.2.1). Hierarchical clustering analysis of Bray-Curtis distances was conducted to evaluate differences in composition and clustering of soil and CW gut microbial communities. The UCLUST function of the STAT package for R was used to perform clustering using default parameters and the unweighted pair group mean average (UPGMA) algorithm. The R package ggtree was used for dendrogram visualization [42]. To compare the effects of plant clones with TS content on the soil and adult weevil gut microbial communities, volcano and Manhattan plots were used to visually assess differential ASVs according to methods described by Zgadzaj (2016) [43].

### Source-tracking of the CW gut microbiome populations and isolation of bacteria functional for toxin degradation

A source model of microbiome (SMM), the conceptual model of the plant microbiome source, was constructed based on possible sources of microbial populations and interactions among CW gut, soil, and fruit microbial communities. Specifically, the Source Tracker software [44] program and its associated Bayesian algorithm were used to predict the proportion of sink samples from each source based on the community structures of source and sink samples. The gut, soil, and fruit microbiome community data were used as sources once, and the remaining two groups of data were used as sinks five times each calculation.

After identifying the gut microbiome being highly influenced by soil microbiomes, we attempted to isolate and identify key TS-degrading bacterial taxa. Solid medium with TS as the single carbon source was used to screen for bacterial isolates that could degrade TS. Media (per L) included 5.00 g TS (Purity: 98%, China), 5.00 g (NH_4_)_2_SO_4_, 2.50 g Na_2_CO_3_, 0.30 g KH_2_PO_4_, 0.05 g FeSO_4_·7H_2_O, 0.50 g MgSO_4_, and 16.00 g agarose in 1 L of distilled water (pH 7.2). Soil solutions and gut homogenates were diluted across gradients of 10^−1^ to 10^−7^. Then, 2 uL of the diluted solutions were uniformly coated on TS medium plates and cultured at 37 °C for 24 h to observe bacterial growth. Single bacterial colonies were selected and grown at 37 °C, shaking at 200 rpm for 12 h followed by DNA extraction PCRs were conducted using the extracted DNA and the 16S rRNA gene primers 8F (5′-AGAGTTTGATCCTGGCTCAG-3′) and 926R (5′-CCGTCAATTCCTTTAAGTTT-3′). The obtained 16S rRNA gene sequences were compared against the NCBI database to evaluate taxonomic identity in addition to the construction of Maximum Likelihood and Bayesian Inference phylogenetic trees using the 16S rRNA gene data. The strains AS23 and T4 of *Acinetobacter* sp. were further subjected to whole genome shotgun (WGS) sequencing on the Illumina NovaSeq and PacBio Sequel sequencing platforms (Bio-Project ID of bacteria genome sequencing raw data: PRJNA785292). The Step MCScanX software package from TBtools was used to analyze the collinearity of the two genomes [45].

### Metagenomic analyses

To better understand the metabolic pathways associated with TS degradation, metagenomic sequencing was conducted on soil and gut microbiome community samples along saponin degradation course. Soil was first treated with an aqueous solution containing 5 g/L of TS. In addition, newly emerged CW adults were fed 5 g/L of TS in an aqueous solution. Community samples were taken at 12 h intervals. Soil samples were taken in triplicate for each time point, while gut samples were taken for nine pairs of adult worms at each time and then mixed into triplicate samples. The experiment was conducted with samples taken from six time points (60 h). Whole genome shotgun sequencing was used on total extracted metagenomic DNA from the samples after fragmenting the DNA. DNA was sequenced on the Illumina Novaseq/Hiseq HTS platforms with 150 bp paired end (PE) libraries, yielding an average of 12 Gbp per sample. Demultiplexed metagenome raw data are available in the NCBI Sequence Read Archive (Bio-Project ID: PRJNA777380). Clean sequence data were obtained by using Cutadapt (v1.17) to filter the data. Species annotation was conducted using Kraken2 [46] and the Megahit software was used for assembly, with contigs > 200 bp being retained [47]. Species annotation information for contig sequences was integrated with the abundance tables for each sample to obtain overall species abundances at each taxonomic rank (domain, phylum, class, order, family, genus, and species) for each community. The MetaGeneMark software (http://exon.gatech.edu/GeneMark/) [48] program was used to identify open reading frames (ORFs), predict coding sequences, and obtain protein annotations. The non-redundant protein sequence sets were compared against the Kyoto Encyclopedia of Genes and Genomes (KEGG) database to further annotate gene functions among samples. The LEfSe analysis software program was used to analyze the abundances of KEGG functions among communities, while ggTree and other R packages were used to visualize the results [49].

To better understand the pathways of TS degradation within soil and CW gut microbiomes, bacterial genomic binning assembly was performed from the metagenomic data. Network analysis was conducted using the bin information with the Psych R package (version 4.0) [41], followed by visualization with GEPHI [50]. Spearman correlations were calculated, and statistically significant correlations (*p* < 0.001) were retained for further analysis. In addition, Simpson, Chao1, and Shannon diversity indices were calculated in R (version 4.0) [41] to compare the changes in bacterial genome diversity in the soil and larval guts treated with TS. To clarify the role of *Acinetobacter* in TS degradation, *Acinetobacter* genomes were annotated from the binning analysis and annotated against the KEGG pathway database. R (version 4.0) [41] was again used to analyze the correlations among the data, as visualized with a Sankey diagram [41].

### Degradation of TS by Acinetobacter sp. AS23

To further clarify the function of strain AS23 of *Acinetobacter* sp. in degrading TS, experiments were carried out with TS as the single carbon source within medium. Liquid medium was prepared using 5 g/L TS and the rest as described above. Each treatment group was cultured at 37 °C by adding 20 μL of a single bacterial solution with an OD value of 2.0 into 100 ml of medium, shaking at 200 rpm. The control group (CK) was cultured with 20 μL of sterile water under the same conditions. Samples were aseptically taken every 12 h, with 1 mL of solution, from each culture for saponin detection and repeated with five samples at each time point. Samples from 12, 36, and 60 h time points were used to measure residual TS content.

To further verify the effects of the strain AS23 on degradation TS in CW guts, 30 larvae were treated with gentamicin sulfate, tetracyclines, and rifampin. After 24 h, cultured AS23 cells were mixed with sterile honey water and fed to larvae. Thirty larvae were divided into five groups and placed in 5 g/L of TS fodder. After feeding for 7 days, all the larvae were removed, and feces were used to evaluate TS content.

### Determination of TS content

TS content was determined as described by Zhang et al. [21]. Briefly, an Agilent Eclipse XDB-C_18_ (4.6 mm × 250 mm, 5 μm) high performance liquid chromatography (HPLC) instrument was used for quantification. One milliliter of fermentation liquid was drawn up, dissolved in ultrasonic methanol, and placed in a 10-mL volumetric flask. The results were analyzed using HPLC after 0.2 μm microporous membrane filtration. The mobile phases were methanol-water (*V*_methanol_:*V*_water_ = 9:1) and the detection wavelength was 210 nm, while the column temperature was set to 25 °C. Standard treatment included 0.05 g of TS standard that was accurately weighed (with an accuracy to 0.0001 g) dissolved ultrasonically in methanol and placed in a 10 mL volumetric flask. Then, transferring of a gradient of 1 mL to 9 mL of methanol was conducted to establish a gradient dilution from 10^−1^ to 10^**−**7^, with volume measurement in a 10 mL volumetric flask. A 0.2-μm microporous membrane was used for analysis after filtration. Mass concentrations were calculated from the abscissa and the corresponding peak area as the ordinate. Standard curves were drawn with the regression equation calculation.

### In vivo assays of AS23 colonization

To further investigate the saponin-degrading functions of AS23 strain, PBBR-GFP plasmid was transferred into the strain as described by Zhang et al. [28]. Larvae were collected and reared under controlled conditions (i.e., in the dark, soil temperature of 20 °C, and moisture of 15%) for the experiment. A total of 280 mature larvae were selected, and 275 were treated with gentamicin sulfate (0.05 g/L), tetracyclines (0.05 g/L), and rifampin (0.05 g/L), while five were fed with sterile water droplets as the control group. After 24 h, the five control larvae and five treated larvae randomly selected from the 275 in the treatment group were collected, their intestinal DNA was extracted, and amplified using *Acinetobacter* specific primers (ACI381F: 5′-CACAATGACATTGCAAGCAATTG-3′ and ACI382R: 5′-CCAATTTTCATACGAATCTGG-3′) [51]. PCR amplification success was evaluated based on the presence of target bands in gel electrophoresis. After ensuring that all *Acinetobacter* within the remaining 270 larvae guts were killed, they were divided into three groups. In the first treatment group (SS), 90 larvae were placed in sterile soil to pupate. In the second treatment group (US), 90 larvae were placed in unsterilized soil to pupate. In the third treatment group (SSA), 90 larvae were placed in sterilized soil mixed with fluorescently labeled AS23 cells to pupate. All treated groups were incubated at room temperature under aseptic conditions for subsequent experiments. After the emergence of adults, three pairs of adults were taken from each treatment group for gut dissection. Presence of fluorescence was detected using a Carl Zeiss Microscope GmbH (Germany).

Twenty-six pairs of adult weevils were randomly selected from the SS, US, and SSA treatments. The *C. oleifera* clone CL 166 that exhibited the strongest resistance to pests was selected to evaluate TS tolerance of larvae. The quantity of fruit was relatively consistent across plants. To prevent CW adults from escaping, the *Camellia* tree was covered with a transparent plastic net in advance. A pair of CW adults were randomly selected from each control and gut fluorescence treatment and all guts were subjected to detection of fluorescence. Five pairs of CW adults selected from each treatment group were also freely reared on a *C. oleifera* tree to allow mating and oviposition, and five replicates were taken from each treatment group. The fruits and larvae were recovered for the first time on June 20, 2021. The fruits from one *Camellia* tree were retrieved from each treatment group. All fruits were cut open, and the developing larvae were extracted from the fruits and synchronously weighed to verify the effects of TS on their development.

## Results

### Camellia weevil gut microbiomes differ from surrounding environmental microbiomes

A total of 270 microbial community samples from CW larvae guts, *C. oleifera* fruits, and soil (*n* = 90 each) were subjected to HTS of 16S rRNA genes, with between 147,592 and 68,784 sequence reads obtained per sample. After quality filtering, between 136,292 and 62,982 high quality sequence reads were obtained per sample. The sequence reads represented a total of 233,731 non-singleton amplicon sequence variants (ASVs). Unconstrained principal coordinates analysis (PCoA) of Bray-Curtis distances among communities indicated the presence of three distinct sample clusters corresponding to sample type (Fig. 1d and Table S1). The soil and fruit samples partially overlapped in space along the first axis, while soil and gut communities partially overlapped along the second axis (Fig. 1d). Variation among the three types was statistically significant based on *Anosim* test (*R* > 0.5, *p* < 0.05) and nonparametric multivariate analysis of variance test (*Adonis*, *p* = 0.001) (Fig. 1d). Nevertheless, these data did not necessarily indicate that there exist complete differences in taxa among the soil, fruit, and larvalgut microbiomes (Fig. 1d and Table S2). To explore whether sampling regions potentially had influence on microbiome difference, a second PCoA was visualized. The differences (*Anosim*, *R* < 0.5, *p* < 0.05) in soil and gut microbiomes collected from different locations (Figure S2 and Table S3 and S4) were minor. In contrast, highly significant differences were observed for microbiomes of fruits collected from different regions (*Anosim*, *R* > 0.75, *p* < 0.05), indicating that fruits were more susceptible to external different environments (Figure S2 and Table S3). All seven evaluated alpha diversity indices exhibited statistically significant differences among types (Welch’s *t* test, *p* < 0.05) (Figure S1b). Species diversity and richness of gut microbiomes were lower than those of soil and fruit samples (Figure S1b).

Proteobacteria was the most dominant phylum among all three sample types (Figure S1a). Further, Proteobacteria comprised roughly 65% of the soil and fruit microbiomes (Figure S1a). Firmicutes exhibited higher abundances (22.26%) in fruit sample microbiomes (Figure S1a). Soil microbiomes were relatively even, wherein the proportions of other bacteria phyla were relatively consistent across samples, except for Proteobacteria (Figure S1a). After excluding ASVs that could not be annotated to the genus, *Acinetobacter* (46.14%) and *Aquabacterium* (25.40%) accounted for the highest abundances in the soil microbiomes (Figure S1a). *Aquabacterium* (49.89%) was the most abundant genus in the fruit samples (Figure S1a). In contrast, the most abundant genus in the CW gut microbiomes was *Serratia*, followed by *Acinetobacter*. Random forest machine learning classification was used to identify discriminatory taxa for soil, fruit, and gut microbiomes at the genus level, using 10-fold cross-validation that was repeated five times to evaluate classification accuracy (Fig. 1e).

A ternary analysis was used to assess the relationship between weevil gut microbial populations and the microbiomes of their surrounding environments (e.g., soil and fruits). Only a few ASVs (3.39%) were specific to soil (327 total ASVs), fruits (2,264 ASVs), and guts (4260 ASVs). *Methylobacterium*, the 1174-901-12 group, *Burkholderia*, *Lactobacillus*, and *Aquabacterium* were most enriched in fruit microbiomes (Fig. 1e), while *Aquabacterium*, *Serratia,* and *Acinetobacter* were predominant in soil microbiomes (Fig. 1e). Noticeably, *Serratia*, *Acinetobacter*, and *Enterobacter* were significantly enriched in weevil gut microbiomes. Thus, *Serratia* and *Acinetobacter* were both identified as core components of gut and soil microbiomes, while *Aquabacterium* was important within fruit and soil microbiomes (Fig. 1e). Moreover, fruit and gut microbiomes exhibited closer similarities to soil, while soil exhibited fewer overall unique ASVs (Fig. 1f). Only 10 most abundant genera exhibiting the highest importance to the overall model were selected as potential biomarker (Fig. 1g). A comparative analysis of microbiome diversity and structure indicated that gut microbiomes differed from those in fruits and soil, and they were affected by the environment, in addition to many common ASVs identified from soil and gut samples (Fig. 1f, g).

### Gut microbiome source tracking and exploration of common culturable bacteria

**Fig. 2:**
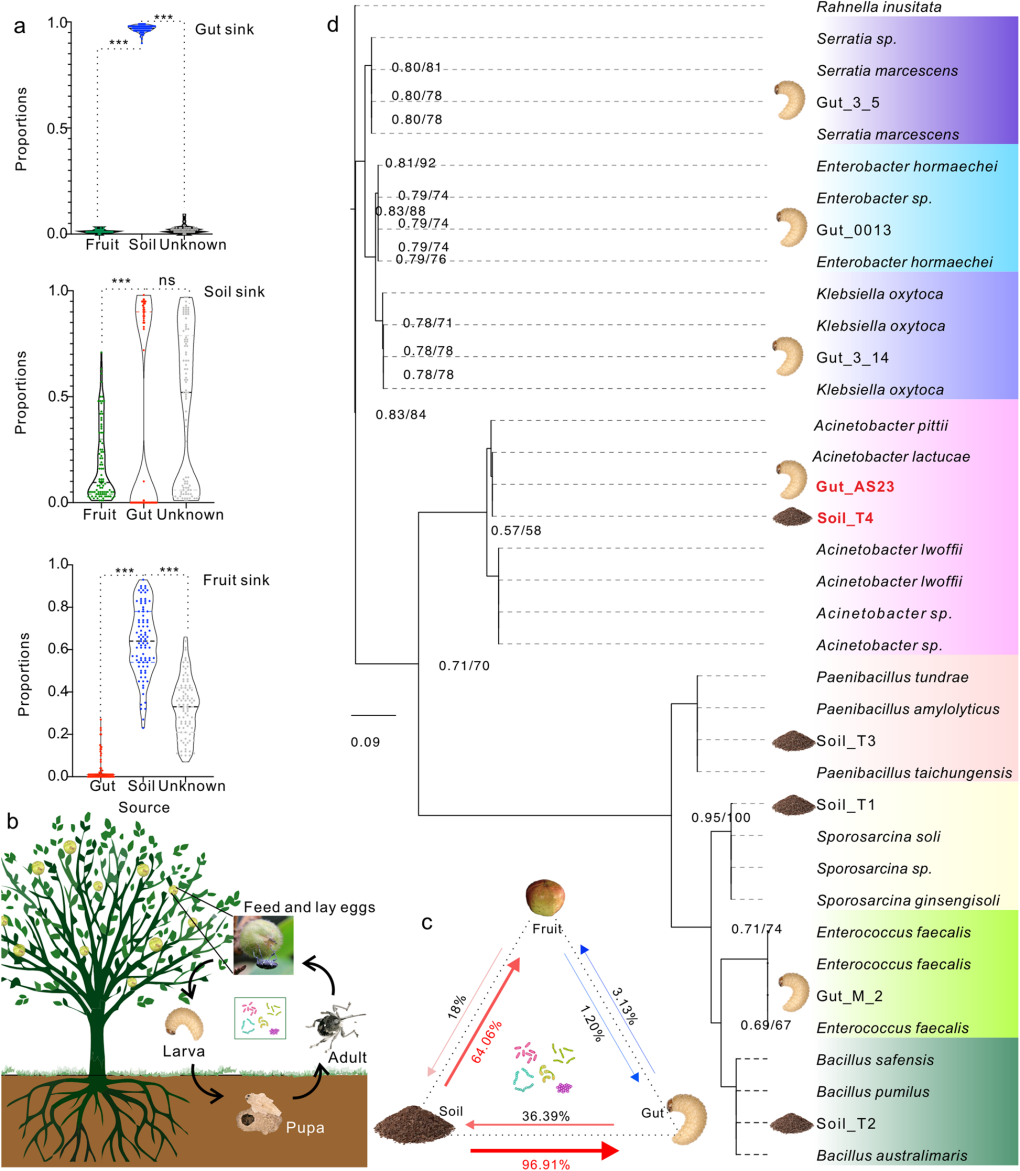
Source-tracking analysis of gut bacterial populations and the phylogenetic tree reconstruction of culturable isolate 16S rRNA genes. **a–c** Source model of microbiome (SMM) showing the possible sources of gut-associated bacterial communities based on all three sample types (*n* = 270). **a** Source-tracking analysis graph that represents predictions of sources, with colored violin plots representing the proportion of each source in a sample. Unknown indicates classification of unknown sources and error lines indicate the standard deviation of 100 Gibbs samples. ‘***’ was used to identify statistical significance (*p* < 0.05). **b** Schematic showing that CW complete its life cycle on a *C. oleifera* tree. **c** Results of microbiome source tracking analysis among the three types of samples. The arrows point from the source microbiome to the affected microbiome and the color of the arrow represents the degree of influence. The thickness of lines is equivalent to the source contribution. The red arrow indicates significance test *p* < 0.05, while the blue arrow indicates insignificance. **d** Phylogenetic tree reconstruction of culturable strain 16S rRNA genes. The Maximum Likelihood (ML) and Bayesian Inference (BI) trees exhibited the same topological structure, and the support of nodes is expressed by ML/BI bootstraps. Only node supports of > 0.55/55 are shown in the visualization

Gut microbial source tracking analysis was conducted to better understand the influence of surrounding environment on gut microbiomes. A source model of microbiomes (SMM) was constructed for the analysis and the result revealed that soil microbiomes were the biggest source affecting the gut microbiomes of newly emerging CW adults. In contrast, gut microbiomes were slightly affected by fruit microbiomes (Fig. 2a, c). A schematic diagram and conceptual model were developed to mimic the entire life cycle of CW based on the above results (Fig. 2b). Using the model, the adults can encounter external microbial populations only in the stage when the adults emerged from the soil and feed on fruits (Fig. 2c). Again, source tracker analysis indicated the biggest source of gut microorganisms in adults after emergence was soil (96.91%), while fruits were estimated to the contribution of 1.2% to gut microbiomes (Fig. 2c).

After excluding the effects of fruits on CW gut microbiomes, in vitro cultures of bacteria from weevils were established from the soil of highly pest-resistant plant clone roots and the gut microbiomes of adults feeding on clone CL166 fruits. Cultures were established with TS as the only carbon source to encourage isolation of saponin-degrading taxa. Five bacterial species (*Serratia* sp. [strain_3_5], *Enterobacter* sp. [strain_0013], *Klebsiella* sp. [strain_3_14], *Acinetobacter* sp. [strain_AS23], and *Enterococcus* sp. [strain_M_2]) were cultured from the guts, while four species (*Acinetobacter* sp. [strain_T4], *Paenibacillus* sp. [strain_T3], *Sporosarina* sp. [strain_T1], and *Bacillus* sp. [strain_T2]) were cultured from soil (Fig. 2d). Intriguingly, in vitro culture results produced the same results for taxonomic overlap as the ternary plot visualizations and random forest classification analysis (Fig. 1e–g). Among the above cultured genera, *Acinetobacter* exhibited the most common abundances in guts and soil (Fig. 1e–g). Genomic analysis of the two *Acinetobacter* strains from guts and soil (in addition to subsequent collinearity analysis) indicated that they were completely the same, and no differences were observed at the genomic level (Figure S3).

### Effect of the TS content on CW gut and soil microbiomes

**Fig. 3:**
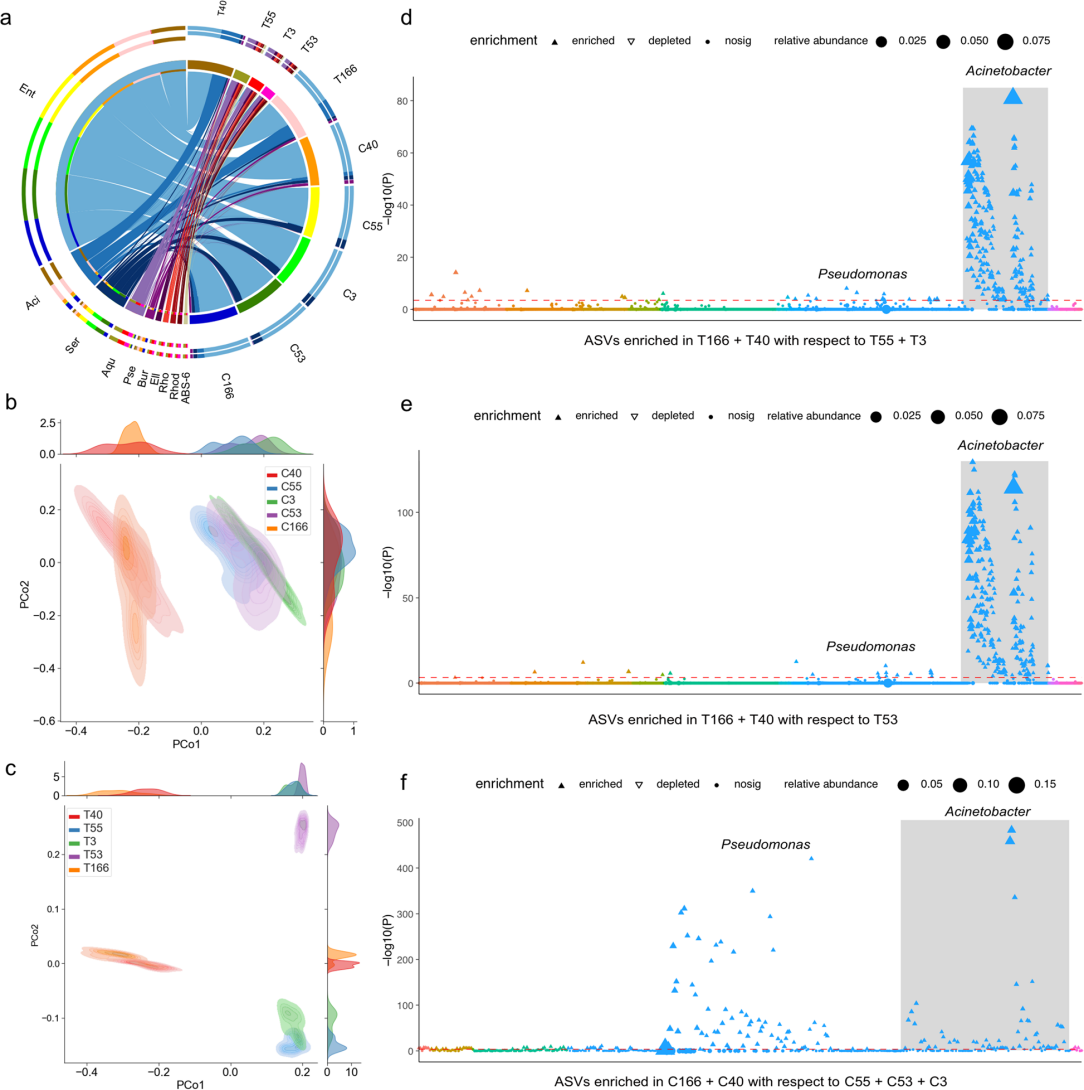
Differences in soil and gut microbial community structures when reared on *C. oleifera* clone plants with different tea saponin contents. Soil samples from the roots of different clones: T3, T40 T53, T55, T166. Gut samples from the larva feeding on different clones: C3, C40 C53, C55, C166. **a** Circle diagram showing the ten most abundant bacterial genera identified in weevil guts and soil. Ent: Enterobacteriaceae, *Aci*: *Acinetobacter*, Ser: *Serratia*, Aqu: *Aquabacterium*, Pse: *Pseudomonas*, Bur: *Burkholderia*, Ell: Ellin6513, Rho: Rhodospirillaceae, Rhod: *Rhodoplanes*, ABS_6: ABS_6. **b** Unconstrained PCoA ordination of Bray–Curtis distances showing that tea saponin content clearly corresponds to distinct gut microbiome samples (*p* = 0.001, *PERMANOVA* test and *Anosim* test). **c** PCoA ordination of Bray-Curtis distances showing that tea saponin content corresponds to a clear division of soil samples into three groups (*p* = 0.001, *PERMANOVA* test and *Anosim* test). **d–f** Manhattan plots showing enriched ASVs in the T166 + T40 group with respect to the T55 + T3 and T53 groups, in addition to those enriched in C166 + C40 with respect to C55 + C53 + C3. ASVs that are significantly enriched (also with respect to soil) are depicted as full circles. The dashed lines correspond to the false discovery rate-corrected *p* value threshold of significance (*p* < 0.05). The color of each dot represents the different taxonomic affiliation of the ASVs at the order level, while the size corresponds to their relative abundances in their respective samples. Gray boxes are used to denote ASVs which were identified as *Acinetobacter*

The bacterial taxa that mediate CW mitigation of TS toxicity were investigated, presuming that soil microbiomes are likely the significant source for gut microbiomes. First, the soil and gut microbiomes were regrouped based on plant clones (Fig. 3a). PCoA analysis indicated that gut microbiomes were completely divided into two clusters along the first axis (Fig. 3b). Further, soil microbiomes were clearly divided into three clusters along the second axis (Fig. 3c). These results suggest a possible correlation between microbiome composition and TS content among plant clones. The relative abundances of *Aquabacterium* were highest in root soil samples from plant clones of T55, T53, and T3 (soil samples from the roots of different clones: T3, T40 T53, T55, T166.) with low TS content (Fig. 3a). In contrast, the relative abundances of *Serratia* were highest in the gut microbiomes (C55, C53, and CC3) of the adults fed on fruits of low TS content plant clones (Fig. 3a). Hierarchical clustering analysis of microbiomes corroborated the clustering of samples observed in the PCoA ordination (Figure S4) and species composition results were completely consistent with the results of circle diagram analysis (Figure S4). Both *Serratia (*nutrient metabolism) and *Acinetobacter* (toxic degradation) were enriched in gut microbiomes likely due to high TS content (Fig. 3a and Figure S4). Noticeably, *Acinetobacter* was significantly abundant in the roots of plants that had high saponin contents in soil (Fig. 3a and Figure S4).

Further analysis revealed differences between soil microbiomes collected from high TS content roots (T166 + T40) and low TS content roots (T55 + T3 and T53) that could be divided into three groups in a PCoA ordination (Fig. 3d, e). Differential community analysis of T166 + T40 samples and T55 + T3 samples revealed that 5594 ASVs were significantly enriched in T166 + T40 samples (Figure S5). Comparison between T166 + T40 and T53 revealed that 8266 ASVs were significantly enriched in the T166 + T40 samples (Figure S5). The clones CL40 and CL166 with high TS content led to enrichment of many soils microbial taxa including Proteobacteria (*Pseudomonas*, *Acinetobacter*, *Aquicella*, *Pseudomonas*, *Cronobacter*, and *Alkalimonas*) and Firmicutes (*Enterococcus*, *Clostridium*, *Phenylobacterium*, *Asticcacaulis,* and *Brevundimonas*) (Fig. 3d–f). A total of 75 ASVs were significantly enriched in the gut microbiomes of weevils fed with high TS plants compared with other samples (Figure S5). These ASVs primarily belonged to the Proteobacteria (*Pseudomonas*, *Acinetobacter*, *Pseudomonas*, *Cronobacter*, *Burkholderia,* and *Ochrobactrum*) (Figure S4a and Fig. 3f). Correlational analysis was used to assess whether specific taxa abundances were associated with TS content. The result revealed a positive correlation of *Acinetobacter* abundance with TS content (*R* = 0.53, *p* = 2e^−14^) and a significant negative correlation of *Burkholderia* abundance with TS content (*R* = − 0.32, *p* = 8.7e^−6^) (Figure S6).

### Changes in soil and gut microbiome structures and functions due to TS degradation

**Fig. 4:**
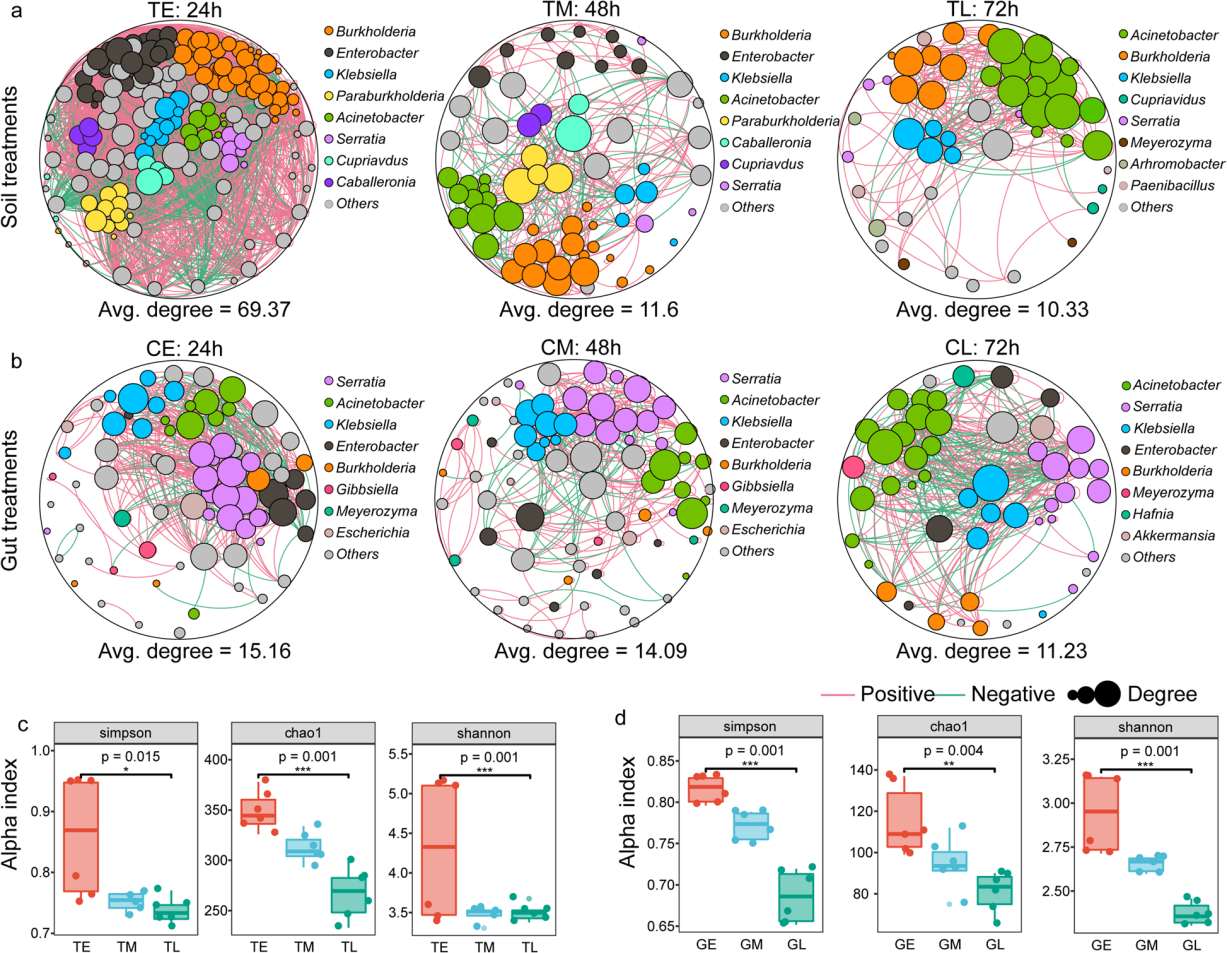
Metagenomic analysis of changes in soil and gut microbial communities during saponin degradation. TE, TM, and TL refer to tea saponin treated soil for 24 h, 48 h, and 72 h respectively. CE, CM, and CL substituted tea saponin feed for larvae for 24 h, 48 h, and 72 h, respectively. **a** Changes in soil microbiome composition after treated with tea saponin over 72 h. **b** Changes in weevil gut microbiome composition after treated with tea saponin over 72 h. Red lines show positive correlations and green lines indicate negative correlations. The area of the circle represents the degree, which is calculated according to bacterial abundances. Only correlations with *R* > 0.6 or < − 0.6 and *p* < 0.05 were included in the networks. The sizes of the nodes are proportional to the number of connections (i.e., the degree). **c**, **d** Bacterial alpha-diversity values of soil and gut microbiome communities during tea saponin degradation

To characterize the changes in soil and gut microbiome structures associated with TS degradation, we assessed the alpha-diversity and co-occurrence patterns of bacterial populations among sample communities. TS content could strongly affect bacterial diversity (i.e., Shannon and Simpson indices, and Chao1 richness) and network complexity (i.e., higher average degrees representing greater network complexity) (Fig. 4a–d). Bacterial richness and network complexity gradually decreased extending from the TE (24 h, with an average degree of 69.37) to TM (48 h, with an average degree of 11.6) and then to TL (72 h, with an average degree of 10.33) communities (Fig. 4a, c). Thus, the influence of TS content on soil microbiomes was relatively severe, wherein average degree dramatically decreased within 48 h (Fig. 4a). Meanwhile, the core genera identified as important network hubs in soil were also significantly affected by TS content (Fig. 4a). Specifically, the abundance of *Acinetobacter* increased with TS degradation time, and it became the primary bacterial taxa in the later stages of TS degradation (Fig. 4a). In addition, larval gut microbiomes responded clearly to saponin degradation time. Bacterial richness and network complexity gradually decreased from CE (24 h, with an average degree of 15.16) to CM (48 h, with an average degree of 14.09), and then to CL (72 h, with an average degree of 11.23) (Fig. 4b, d). Further, the taxonomic composition of the networks differed between CL and the other two, with more nodes belonging to *Acinetobacter* in the former (Fig. 4b). Interestingly, members of the *Acinetobacter* genus were identified as important network hubs in gut microbiome networks that were not significantly affected by TS, although the proportions of *Acinetobacter* changed in these communities (Fig. 4b).

**Fig. 5:**
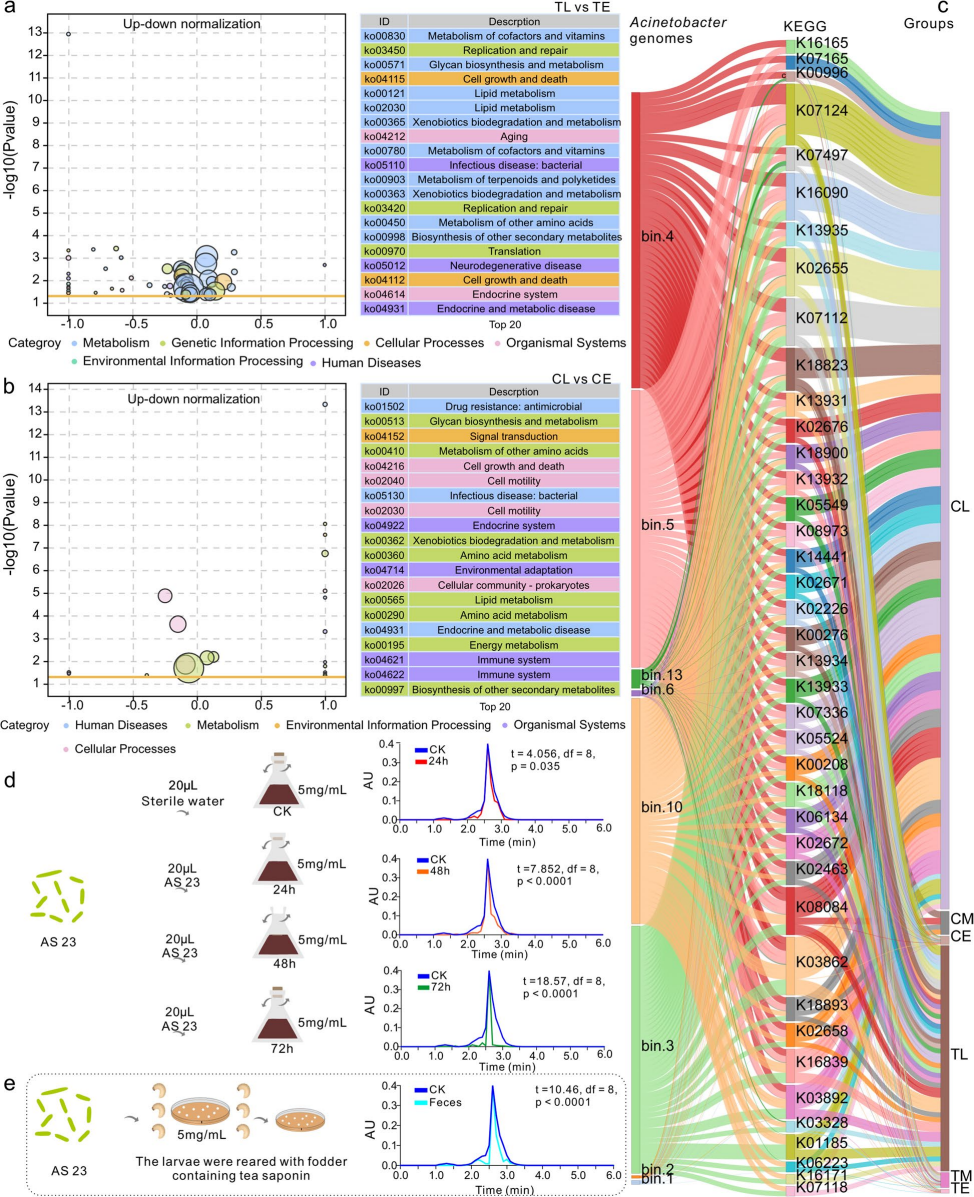
Function and validation of *Acinetobacter* based on metagenomic analysis during tea saponin degradation process. TE and TL refer to tea saponin treated soil for 24 h and 72 h respectively. CE and CL substituted tea saponin feed for larvae for 24h and 72h, respectively. **a**, **b** Top 20 KEGG pathways showing significant differences in abundances when comparing the beginning and end of saponin degradation experiments in soil (**a**) and gut (**b**) samples. The larger the ordinate, the smaller the *p* value, indicating a more significant effect. The abscissa represents the proportion of up-down normalization (the difference between the number of up-regulated and down-regulated genes among the total differential genes). The more right-shifted this value is, the greater the difference between upregulated and downregulated genes enriched within the specified pathway, and the larger the number of upregulated genes that are present within that pathway. The left value indicates the difference between downregulated genes enriched in this pathway is larger than up-regulated genes, and thus, indicates greater downregulated gene numbers. The size of the points indicates gene counts. The orange line represents the *p* = 0.05 threshold. The 20 most abundant pathways are shown on the right, with different colors representing different pathway classes. **c** *Acinetobacter* genomes that could be assembled with genome binning analysis of gut and soil samples and corresponding annotations. The width of the line was determined according to the proportion of read numbers. **d** Verification of tea saponin degradation by *Acinetobacter* sp. (AS23) in vitro culture. Liquid medium was prepared using 5 g/L TS and the rest as described above. Each treatment group was cultured at 37 °C by adding 20 μL of a single bacterial solution with an OD value of 2.0 into 100 ml of medium, shaking at 200 rpm. The control group (CK) was cultured with 20 μL of sterile water under the same conditions. Samples were aseptically taken every 12 h, with 1 mL of solution, from each culture for saponin detection and repeated with five samples at each time point. Samples from 12, 36, and 60 h time points were used to measure residual TS content. **e** Verification of tea saponin degradation by *Acinetobacter* sp. (AS23) within weevil guts. Thirty larvae were treated with gentamicin sulfate, tetracyclines, and rifampin. After 24 h, cultured AS23 cells were mixed with sterile honey water and fed to larvae. Thirty larvae were divided into five groups and placed in 5 sg/L of TS fodder. After feeding for 7 days, larvae were removed, and feces were used to evaluate TS content

Pathways that were significantly enriched in soil microbiomes in the late (TL) and early (TE) stages of TS degradation were compared, revealing that the 20 most abundant inferred pathways could be classified into six overall functional groups (Fig. 5a). Among these, 10 pathways involved in metabolism were most differential (Fig. 5a). LEfSe (Linear discriminant analysis effect size) analyses indicated that 23 KEGG pathways were significantly enriched in the later stages of soil microbial degradation of TS. These pathways included the metabolism of cofactors and vitamins; drug metabolism related enzymes, biotin metabolism, benzoate degradation, phenylalanine metabolism, chlorocyclohexane and chlorobenzene degradation, styrene degradation, porphyrin, and chlorophyll metabolism, aminobenzoate degradation, ether lipid metabolism, and nicotinate and nicotinamide metabolism (Figure S7a). Most of these pathways were related to the degradation of toxic substances, the high molecular weight carbon compounds. Similarly, during the later stages of TS digestion, gut microbiome KEGG pathway annotations were dominated by metabolic pathways, 18 of them enriched and most related to the toxin degradation (Figure S7b).

Above results disclosed that *Acinetobacter* was one of the primary bacterial taxa responsible for the later degradation TS in both gut and soil microbiomes. Consequently, KEGG pathways enriched within *Acinetobacter* genomes were more intensively evaluated using Sankey diagrams. A total of 40 KEGG pathways were significantly enriched among *Acinetobacter* genomes that were recovered from the metagenomes, with a large proportion of these pathways also associated with the later stages of TS degradation in guts and soil microbiomes (Fig. 5c, Figure S8 and Table S5). Signaling and cellular processes were the primary KEGG pathways to which the *Acinetobacter* genome proteins were annotated (Fig. 5c, Figure S8 and Table S5). In particular, the biosynthesis of secondary metabolites, carbon metabolism, carbon-carbon lyases, energy metabolism, fatty acid biosynthesis, porphyrin and chlorophyll metabolism, and styrene degradation pathways were all annotated within the *Acinetobacter* genomes. We concluded that *Acinetobacter* populations were involved in the decomposition of high molecular weight carbon molecules (Fig. 5c, Figure S8 and Table S5).

### Acinetobacter sp. strain AS23 degrades TS

As we previously explored the source of gut microbiome, we screened the presence of AS23 strain in CW gut through tea saponin screening medium (Fig. 2d), and there was no difference between the genome of this strain and that of soil-derived T4 strain (Figure S3). Therefore, AS23 was selected as the target strain to explore the degradation function of *Acinetobacter* to tea saponin. Specifically, the peak area for TS in fermentation liquid was 317.148 ± 8.756 AU·S after 12 h, which was significantly different from the control (CK) (*t* = 4.056, df = 8, *p* = 0.035 < 0.05) (Fig. 5d). Further, increased fermentation time led to TS peak area decrease. The residual peak area for TS at 60 h was 223.974 ± 5.528 AU·S, which again significantly varied from that of CK (*t* = 18.570, df = 8, *p* < 0.0001) (Fig. 5d). Moreover, analysis of AS23-inoculated larval guts showed a detectable content of TS in larval feces as 277.944 ± 6.044 AU·S, which also significantly differed from CK (*t* = 10.460, *df* = 8, *p* < 0.0001) (Fig. 5e).

### Acinetobacter sp. strain AS23 mediates larval tolerance to TS toxicity

**Fig. 6:**
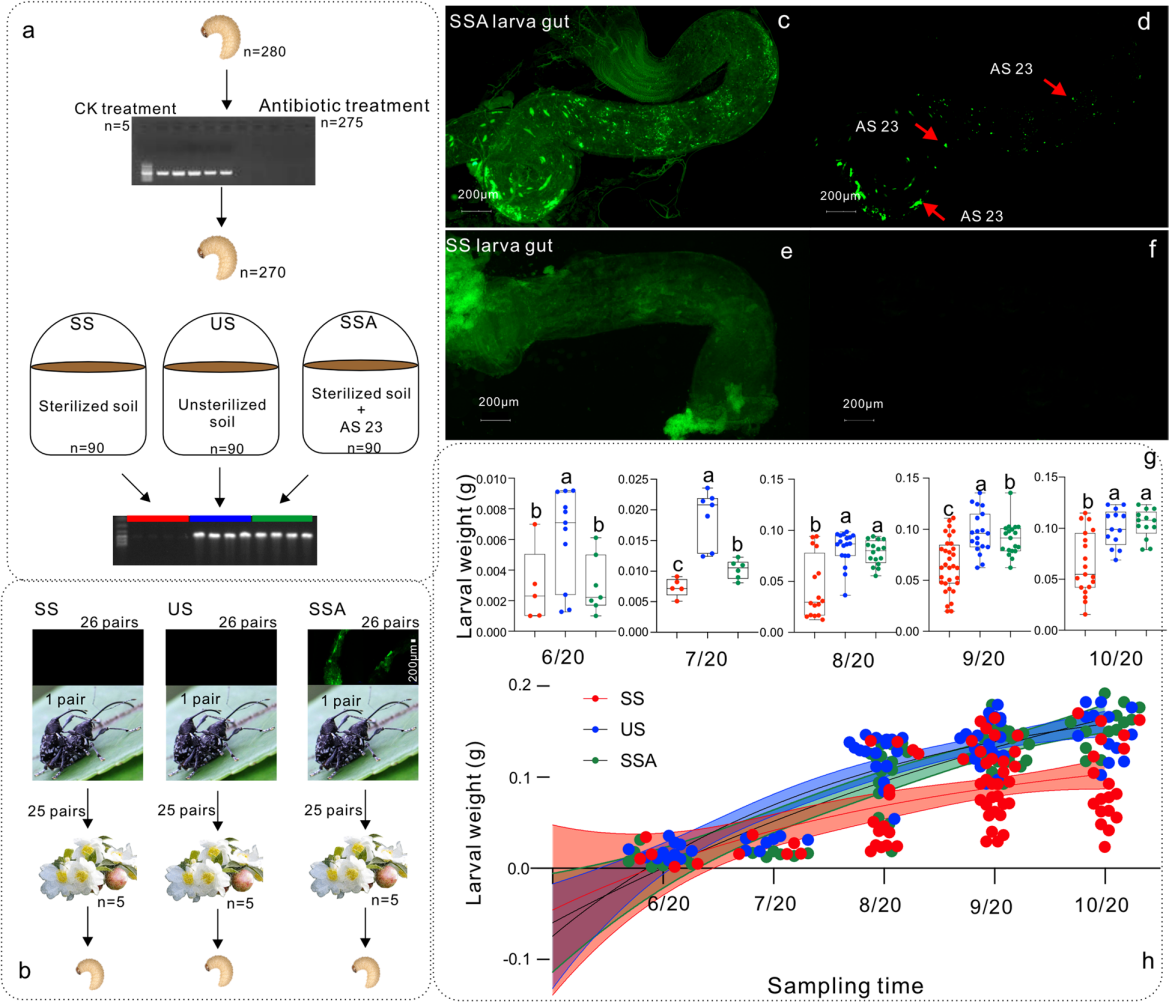
Experimental analysis showing that *Acinetobacter* sp. AS23 from soil mediates the adaptation of CWs to tea saponin toxicity. To further investigate the saponin-degrading functions of AS23, PBBR-GFP plasmid was transferred into the strain as described by Zhang et al. [52]. **a** Schematic showing the treatment of CW larvae in the experiment. Antibiotic treatment: gentamicin sulfate, tetracyclines, and rifampin. CK treatment: sterile water. SS treatment: larvae near pupation were raised in sterilized soil. US treatment: larvae near pupation were raised in unsterilized soil. SSA treatment: larvae near pupation were raised in sterilized soil mixed with fluorescently labeled *Acinetobacter* sp. AS23 cells. **b** CW adults were released on *C. oleifera* trees, and the fruits were regularly collected to evaluate larvae development. **c**, **d** Green fluorescent imaging of AS23 cells (*Acinetobacter* sp. AS23) in the guts of collected larvae and fluorescence imaging of guts. **e**, **f** Fluorescent imaging of guts uninfected with fluorescently labeled *Acinetobacter* sp. strain 23. **g** Comparison of larval weights from individuals obtained at different sampling time. **h** Analysis of larval weight changes from individuals collected at different sampling time. Data were normally distributed, and ANOVA analysis was performed using Prism GraphPad. A *p* value threshold of significance was identified as *p* < 0.05

Five of 275 randomly chosen larvae did not harbor *Acinetobacter*-specific gene sequences after treated with antibiotics, indicating that the antibiotic treatments effectively eliminated *Acinetobacter* from the larvae (Fig. 6a). The remaining 270 antibiotic-treated larvae were placed in three different soil treatments (SS, US, SSA, *n* = 90 each) and allowed to eventually emerge (Fig. 6a). The adult weevils from the US and SSA treatment groups exhibited amplification of *Acinetobacter*-specific gene sequences after emergence (Fig. 6a). In the SSA treated group, fluorescent AS23 cells were present in adult guts after emergence (Fig. 6b). Furthermore, when CW adults fed on fruits and laid eggs, fluorescent AS23 passed through their mouthparts. After egg hatching, larval feeding led to obtaining fluorescently labeled AS23 in the oviposition hole, and thus fluorescent AS23 was detected in larval guts (Fig. 6c, d). In contrast, fluorescence could not be detected in the SS treatment group (Fig. 5e, f). Additional fluorescence analysis indicated that AS23 cells were mainly distributed in larval midguts and hindguts (Figure S9). After hatching, the US treatment group exhibited significantly higher larvae number and weights than the other two groups (SS and SSA) (*p* < 0.05) (Fig. 6g, h). While the number of collected larvae was similar before July 20, the larvae in the US treatment group grew significantly faster than those in the other two groups (Fig. 6g, h). The differences between the SSA and US treatment group larvae then rapidly narrowed with the development of the mature larvae on October 22 which were nearly identical (Fig. 6g, h).

## Discussion

### Camellia weevils acquire microbiomes from soil

The gut microbiomes of insects are highly susceptible to changes due to their surrounding environment [4, 53, 54]. Chewing mouthparts, trace leaves from plant hosts, soil, and even airborne microorganisms could be the sources of gut microbiomes during insect feeding [27, 54]. In particular, the gut microbiota of Hemipterans like aphids and planthoppers are more susceptible to the effects of endophytic bacteria and plant host compounds [6, 26]. *Camellia* weevils (CWs) are a unique group of Coleoptera that exhibit special life stages, mouthparts, and feeding characteristics [21]. The early-stage larva lives completely enclosed within fruits and is almost completely isolated from outside environment [21]. During this stage, larvae possess typical chewing mouthparts, while later stages of larval development exhibit gut microbial structures that are more susceptible to influences from food compounds than plant endophytic bacteria due to enclosed space [21]. With data exploring, we infer that the microflora of larvae mainly derives from female adults transferring microorganisms to eggs during oviposition (Fig. 2b, Fig. 6). Consequently, an understanding of the source of CW adult microbiomes is needed to clarify the source of larval microbiomes. Female adults need to emerge from the soil pupal chamber using their mouthparts, and their gut microbiomes are exposed to soil microbial community before oviposition (Fig. 2b, Fig. 6). When laying eggs, female weevils need put the mouthparts into the oviposition channel in the fruits t, and bacterial transmission may occur (Fig. 2b, Fig. 6). Soil microorganisms are therefore potentially important and can affect the gut microbiomes of CWs.

Significant differences were observed in microbial diversity among fruits, soil, and guts of adults after emergence (Fig. 1d and Figure S1b). Further, soil and adult gut microbiomes after emergence exhibited some similarity in community compositions (Fig. 1d). Nevertheless, many ASVs were specifically enriched in the guts of adults, while more ASVs were shared by soil and gut microbiome communities than gut communities with fruits (Fig. 1f and Figure S1c). Random forest classification of indicator species also suggested the presence of some common indicator microorganisms between soil and gut communities (Fig. 1e). Moreover, source tracking analysis indicated that soil microbiomes could significantly influence the gut microbiomes of CWs, exhibiting a probability of gut microbiomes from soil of up to 96.91% (Fig. 2a, c). The results suggest that adult CW gut microbiomes were considerably influenced by soil microbiomes during the emergence process. In addition, the similarities between the microbiomes of fruits and CW guts were very low, with a source tracking contribution from fruits into gut microbiomes of 1.2% (Fig. 2a, c). Therefore, the possibility of fruits affecting the intestinal microbiomes of larvae through oviposition of CWs is low. Importantly, certain bacterial taxa were present both in guts and soil (Fig. 2d). Our subsequent experimental analyses verified that female insects transferred these bacteria to eggs or larvae after being influenced by the soil microbiome (Fig. 6). This was observed and verified using fluorescently labeled bacteria inoculation into soil. After females emerged, fluorescence could be detected in their intestinal regions. Further, fluorescence was detected in larval guts of the offspring that were produced by the females. This suggests that the females transfer bacteria obtained from soil to the larvae during oviposition.

Other studies suggested that intestinal tract flora of herbivorous insects, especially those that coexist in plants and soil, may colonize guts, and played key roles in plant toxin metabolism [4, 27]. For example, some soil microorganisms identified in the guts of *Lespedeza bicolor* proved to endow the host with detoxification for the insecticide fenitrothion [55]. They continuously spread from soil to plant stems and leaves, metabolizing toxins, and thereby creating a dispersal route from plant tissues to the guts of herbivorous insects [27]. Here, we demonstrated that bacterial transmission by female CWs to eggs occurs through the oral cavities from the oviposition channel, indicating another possible route of bacterial transmission from soil to CW gut microbiomes.

### Camellia weevils are protected from TS toxicity by Acinetobacter

The “gut microbiota facilitation hypothesis” suggested that gut microbiomes mediated the adaptations of herbivorous insects to plant host chemical resistances [4]. Microbiota strains have been speculated to degrade or confer tolerance to numerous toxic secondary metabolites from plants including isothiocyanate [14], terpenes [16], alkaloids [29], saponins [21, 56], and polyphenols [56]. *Camellia* weevils are the only herbivorous fruit pest for *C. oleifera* seeds and have co-evolved with their host plants for an extensive period [21]. Consequently, CWs would exhibit a certain capacity for chemical resistance due to their exposure to TS, which is considered as primary defensive compound within *C. oleifera* fruits [21]. This agrees with the previous study indicating that CM gut bacteria may confer inhibition of TS toxicity for the pest [21].

The microbiomes of adult guts and soil samples were highly differentiated when reared on experimentally variable TS contents of plants (Fig. 3b, c). However, both gut and soil communities were highly similar for clones CL 40 and CL 166 with higher contents of TS. It thus considers play an important role in shaping the microbiomes of CWs and soil. Many Firmicutes and Proteobacteria were particularly enriched in the CW gut and soil microbiomes obtained from plant clones with the higher TS content when compared to those treated with low TS content (Fig. 3d−f). Further analysis indicated that only *Acinetobacter* abundances were positively correlated with TS concentration (Figure S6). Moreover, *Acinetobacter* abundances increased with increasing degradation of TS in soil over time, indicating that the bacterium played a key role in TS degradation. Similarly, changes in gut communities after feeding CWs with TS in vivo indicated a gradual change of the core gut microbiota populations from *Serratia* to *Acinetobacter* during the later stages of TS digestion (Fig. 4a, b). These findings indicate that plant secondary metabolites can lead to considerable plasticity in the gut microbiomes of plant-eating insects. They also affect soil microbiomes when entering soil together with litter. Consequently, bacteria associated with the secondary metabolite degradation were significantly enriched in both the gut and soil microbiomes.

Metagenomic analysis revealed that numerous genes involved in pathways associated with the degradation of toxic and high-molecular weight carbon compounds were present in later stages of saponin degradation (Fig. 5a, b). In addition, *Acinetobacter* genomes that were recovered during binning analysis exhibited diverse abundant KEGG pathways related to benzoate degradation, soyasaponin III rhamnosyl transferase, the biosynthesis of secondary metabolites, carbon metabolism, carbon-carbon lyases, energy metabolism, fatty acid biosynthesis, porphyrin and chlorophyll metabolism, and styrene degradation pathways (Fig. 5c). TS structures exhibit hydrophilic and oleophile components [57]. The hydrophilic portion is composed of highly electronegative oxygen-containing groups that are primarily concentrated in the linking components of TS glycosyl ligands, organic acid ligands, and saponin ligands [57]. The main structure of the oleophilic component is a benzene ring structure comprising of a five-ring triterpenoid skeleton with non-polar hydrocarbon ring chains [57]. Benzoate degradation and its related pathways are intermediate in the degradation of phenolic compounds. They include TS degradation of intermediate glycosides that could be further degraded into fatty acids with a terminal end-product, acetyl-CoA. It can then be used in the citrate cycle metabolic pathway [16]. Estradiol dioxygenases involved in aromatic ring cleavage and hydroxylation are important enzymes decomposing aromatics into fatty acids [16]. In addition, phenol can be degraded via the ortho and β-ketoadipase pathway [58]. In present study, soyasaponin III rhamnosyl transferases played important roles in the biosynthesis and degradation of triterpenoid saponins [59]. Pathways corresponding to the above secondary metabolite degradation were identified in the soil and gut community metagenomes of *Acinetobacter* genomes (Fig. 5a–c). Thus, enzymes related to those identified above were likely involved in the degradation of TS (Fig. 5a–c). We therefore infer that *Acinetobacter* is the core bacterium responsible for degrading TS and mitigating the toxicity in CWs.

### Acinetobacter sp. AS23 can degrade TS and allow Camellia weevils to subsist inside fruits

Previous studies disclosed that *Acinetobacter* resistance to toxins derives from plasmids that carry functional genes within strains [60, 61]. Noticeably, the AS23 strain genome identified here did not exhibit an extrachromosomal plasmid (Figure S3), indicating its resistance to TS regulated entirely through its chromosome. *Acinetobacter* is known to degrade toxins or tolerate toxic compounds within host guts and soil [60]. This study also showed that AS23 in liquid medium can use TS as a single carbon source during fermentation, effectively degrading TS (Fig. 5d). In addition, TS in the feces of CWs with *Acinetobacter* sp. AS23 present in their guts had been degraded (Fig. 5e), indicating that AS23 could degrade TS both *in vitro* and within host intestines.

*Acinetobacter* is also frequently associated with aspen foliage and the gypsy moths that infest host plant leaves, and the strain of *Acinetobacter* sp. R7-1 can metabolize phenolic glycosides [30]. Likewise, species of *A. calcoaceticus* and *A. oleivorans* can degrade catechin and modulate host physiology and metabolism to achieve efficient hexadecane utilization [30]. Further, *Acinetobacter* derived from wood-fed termite guts can efficiently degrade phenolic compounds by using phenol as its sole carbon source [30].

The AS23 colonization experiments demonstrated that AS23 primarily colonizes the midguts and hindguts of the larvae (Fig. 6c, d; Figure S8), consistent with the observations in Mason et al. for gypsy moth gut microbiomes [62]. The results also confirmed that CWs can transfer AS23 cells carried by mothers to offspring via oviposition, thereby enabling following generations with the ability of TS degradation. Although the transmission process was not yet fully clear, the detection of fluorescent AS23 cells in offspring confirmed the conclusion. Comparison of the development of offspring produced in the SS, US, and SSA treatments further verified the role of AS23 in helping larvae mitigate TS toxicity (Fig. 6g, h).

The growth of AS23-carrying larvae from the SSA group was not different from those of the SS group initially, which could be related to the relatively simple bacterial gut microbiomes emerging from sterilized soil. However, with the larval development, the growth of the AS23-carrying larvae was gradually like that of the US larva group. This can be explained that TS-degradation AS23 from the intestinal flora gradually became a core functional microbiome leading to less energy expenditure from larval hosts to mitigate saponin toxicity. Thus, we can conclude that AS23 strain plays a key role in the rapid degradation of TS and helps larvae mitigate TS toxicity.

## Conclusion

In this study, comprehensive community profiling methods including 16S rRNA gene high throughput sequencing and metagenomics analyses were used to identify the source of CW gut microbiomes. Soil bacteria were isolated and putatively regarded responsible for the saponin-degrading activity within CW guts. Subsequent experiments using fluorescently labeled cultures verified that the *Acinetobacter* sp. strain AS23 derived from soil, could migrate into CW larval guts, and ultimately endowed its insect host with the ability to degrade toxic saponin, thereby allowing CWs to subsist as a pest inside fruits. These systematic studies of the sources of gut microorganisms, the screening of taxa involved in plant secondary metabolite degradation, and the investigation of bacteria hypothesized to be responsible for CW toxicity mitigation provide conclusive evidence for the intestinal microorganisms mediated tolerance of herbivorous insects against plant toxins.

## Supplementary Information

**Additional file 1: Figure S1.** Species composition and alpha index of the microbiota of samples from soil, fruit, and gut. **a**, Phylum- and genus-level distributions of microbial communities recovered from fruit, soil, and weevil gut microbiota. The relative abundances of taxa that could not be annotated to the genus level are excluded from these plots. **b**. alpha index values for microbial communities from soil, fruits, and weevil guts. The horizontal bars within boxes represent medians. The tops and bottoms of boxes represent the 75th and 25th percentiles, respectively. The upper and lower whiskers extend to data no more than 1.5× the interquartile range from the upper edge and lower edge of the box, respectively. T: Soil, G: Fruit, C: Gut. **Figure S2.** Unconstrained PCoA with bray–curtis distance showing that three sources of the microbiome occur separately from each other (*p* = 0.001, *PERMANOVA* test and *Anosim* test). **a**. All samples were differentiated according to different sources. **b**. Unconstrained PCoA with Bray-curtis distance showing the clustering of soil samples. **c**. Unconstrained PCoA with Bray-curtis distance showing the clustering of fruit samples. d. Unconstrained PCoA with Braycurtis distance showing the clustering of gut samples. **Figure S3.** Collinearity of the *Acinetobacter* sp. genomes from soil and gut. The Step MCScanX software package from TBtools was used to analyze the collinearity of the two genomes. **Figure S4.** Hierarchical clustering analysis of communities from gut (a) and soil (b) microbiomes (based on Bray-Curtis distances) reared on different clone plants. Panel on the left is a hierarchical clustering dendrogram indicating sample similarities. Shorter branch lengths between samples indicate higher similarity between samples. The panel on the right shows a stacked histogram of the 10 most abundant genera. **Figure S5.** Analysis of enrichment difference of ASVs level. **Figure S6.** Correlation between relative abundance of genus level flora and content of tea saponin. **Figure S7.** Functional difference analysis of microbiome during tea saponin degradation. TE, TM, and TL refer to tea saponin treated soil for 24h, 48h and 72h respectively. GE, GM, and GL substituted tea saponin feed for larvae for 24h, 48h and 72h, respectively. **a.** Functional difference analysis of soli microbiome during tea saponin degradation. b. Functional difference analysis of gut microbiome during tea saponin degradation. **Figure S8.** Functional KEGG pathway network of *Acinetobacter* genomes during degradation of tea saponin. **Figure S9.** Green fluorescence imaging of larva guts of the collected larvae and the fluorescence imaging of the gut. a, c, and e, Green fluorescence imaging of AS23 (*Acinetobacter* sp. Strain_23) in the guts of the collected larvae. b, d, and f, AS 23 (*Acinetobacter* sp. Strain_23) GFP luminescence after intestinal tissue autofluorescence was excluded. g, i, and k, Green fluorescence imaging of control group larva gut. h, j, and l, The control group’ gut excluded intestinal tissue fluorescence.

**Additional file 2: Table S1.** Multivariate analysis of variance based on bray–curtis distance among all samples. **Table S2.** Nonparametric multivariate analysis of variance between all samples based on bray–curtis distance. **Table S3.** Multivariate analysis of variance based on bray–curtis distance among soil and gut samples. **Table S4.** Nonparametric multivariate analysis of variance based on bray–curtis distance. **Table S5.** KEGG information annotated by *Acinetobacter* in Binning analysis

## Data Availability

Demultiplexed raw data are available in the NCBI Sequence Read Archive (Bio-Project ID of 16S rRNA gene high throughput sequencing raw data: PRJNA777383; Bio-Project ID of metagenome sequencing raw data: PRJNA777380; Bio-Project ID of bacteria genome sequencing raw data: PRJNA785292).
