# Supplementary material for: Soil-derived bacteria endow Camellia weevil with more ability to resist plant chemical defense
Source: Microbiome. 2022 Jun 25;10:97. doi: 10.1186/s40168-022-01290-3 (PMC9233397; doi:10.1186/s40168-022-01290-3)

Soil-derived bacteria endow *Camellia* weevil with more ability to resist plant chemical defense

Shou-ke Zhang[1,2], Zi-kun Li[2], Jin-ping Shu[3,*], Huai-jun Xue[4], Kai Guo[2], Xu-dong Zhou[1,2,*]

[1]State Key Laboratory of Subtropical Silviculture, Zhejiang A&F University, Hangzhou, Zhejiang 311300, P. R. China.

[2]School of Forestry and Biotechnology, Zhejiang A&F University, Hangzhou, Zhejiang 311300, P. R. China.

[3]Research Institute of Subtropical Forestry, Chinese Academy of Forestry, Hangzhou, Zhejiang 311400, P. R. China.

[4]College of Life Sciences, Nankai University, Tianjin 300071, P. R. China.

*__Correspondence to__: Xu-dong Zhou, School of Forestry and Biotechnology, Zhejiang A&F University, Hangzhou, China. E-mail: xudong.zhou@zafu.edu.cn

Fig. S1 Species composition and alpha index of the microbiota of samples from soil, fruit, and gut.

a, Phylum- and genus-level distributions of microbial communities recovered from fruit, soil, and weevil gut microbiota. The relative abundances of taxa that could not be annotated to the genus level are excluded from these plots. **b**. alpha index values for microbial communities from soil, fruits, and weevil guts. The horizontal bars within boxes represent medians. The tops and bottoms of boxes represent the 75th and 25th percentiles, respectively. The upper and lower whiskers extend to data no more than 1.5× the interquartile range from the upper edge and lower edge of the box, respectively. T: Soil, G: Fruit, C: Gut.

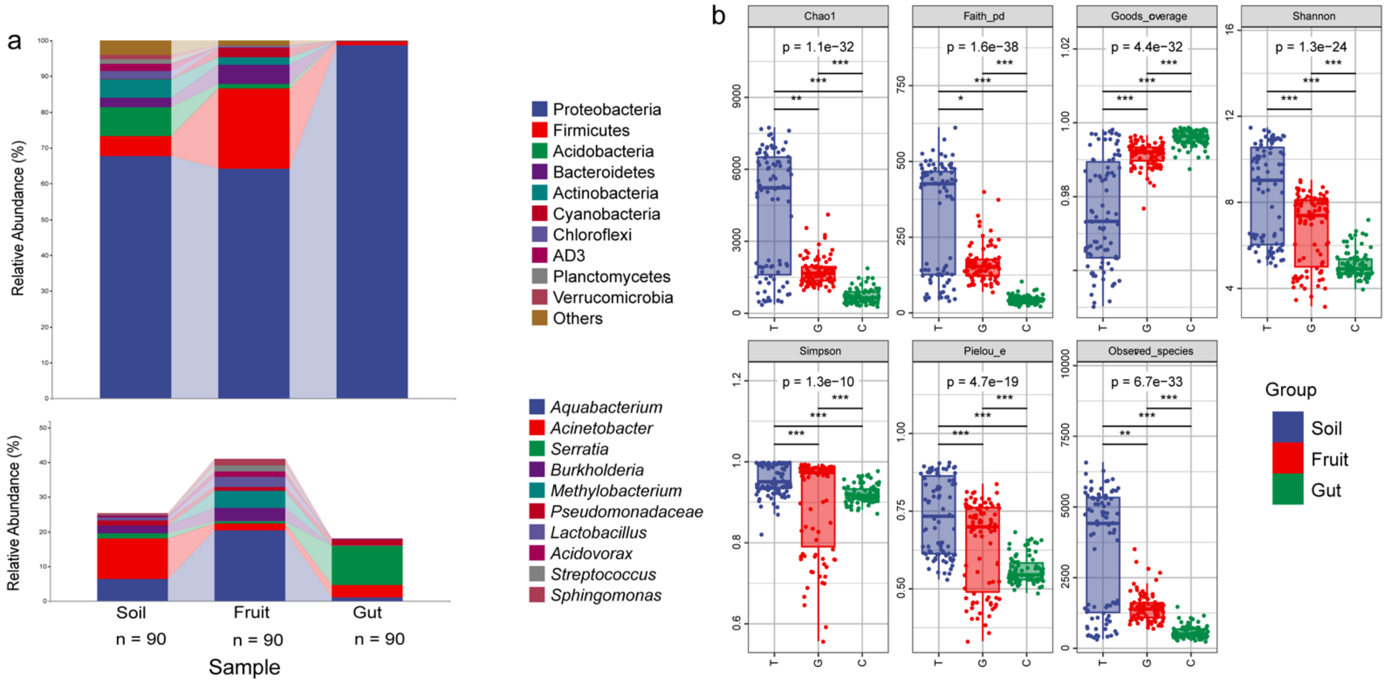

Fig. S2 Unconstrained PCoA with bray–curtis distance showing that three sources of the microbiome occur separately from each other (p = 0.001, *PERMANOVA* test and *Anosim* test). **a**. All samples were differentiated according to different sources. **b**. Unconstrained PCoA with Bray-curtis distance showing the clustering of soil samples. **c**. Unconstrained PCoA with Bray-curtis distance showing the clustering of fruit samples. d. Unconstrained PCoA with Bray-curtis distance showing the clustering of gut samples.

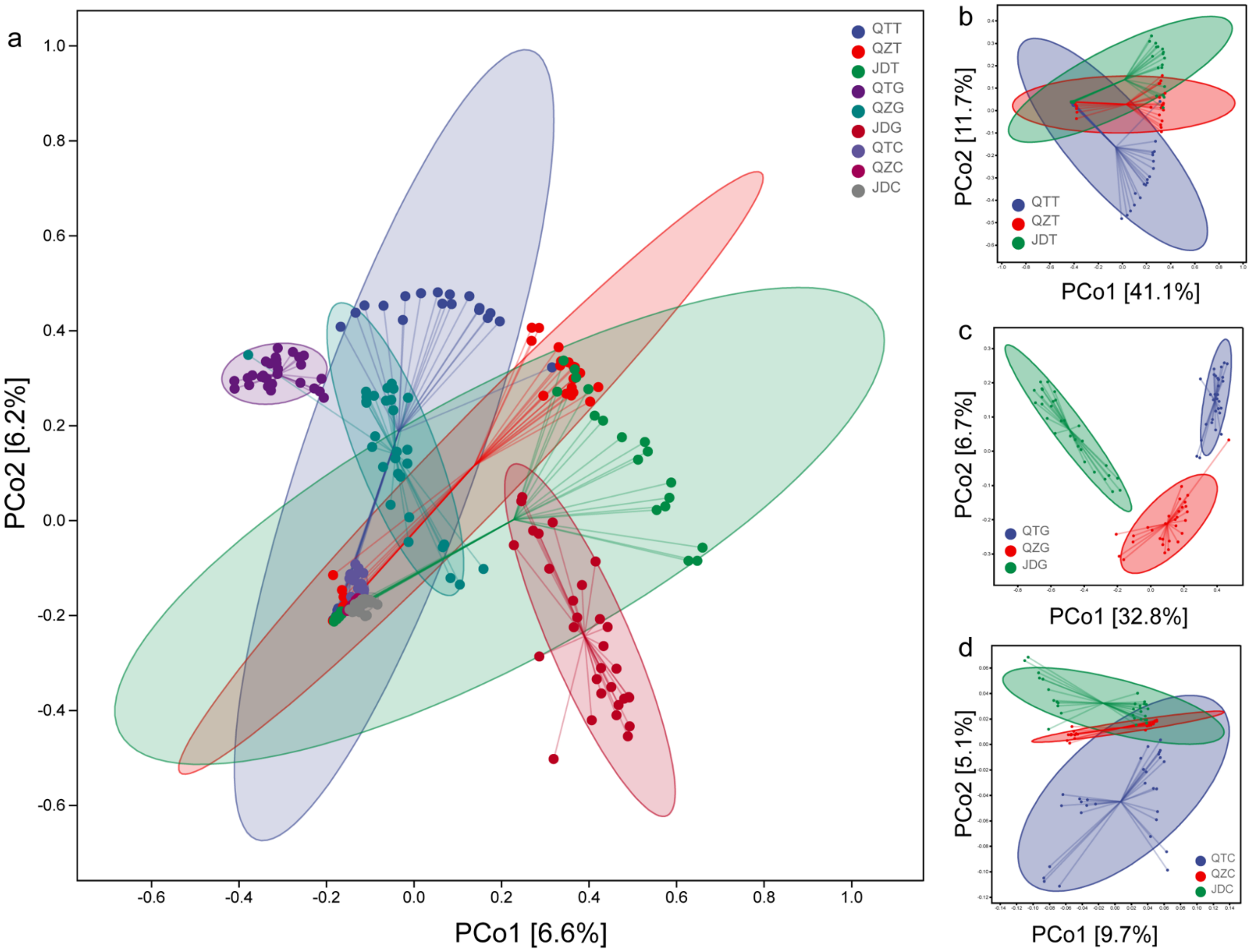

Fig. S3 Collinearity of the *Acinetobacter* sp. genomes from soil and gut.

The Step MCScanX software package from TBtools was used to analyze the collinearity of the two genomes.

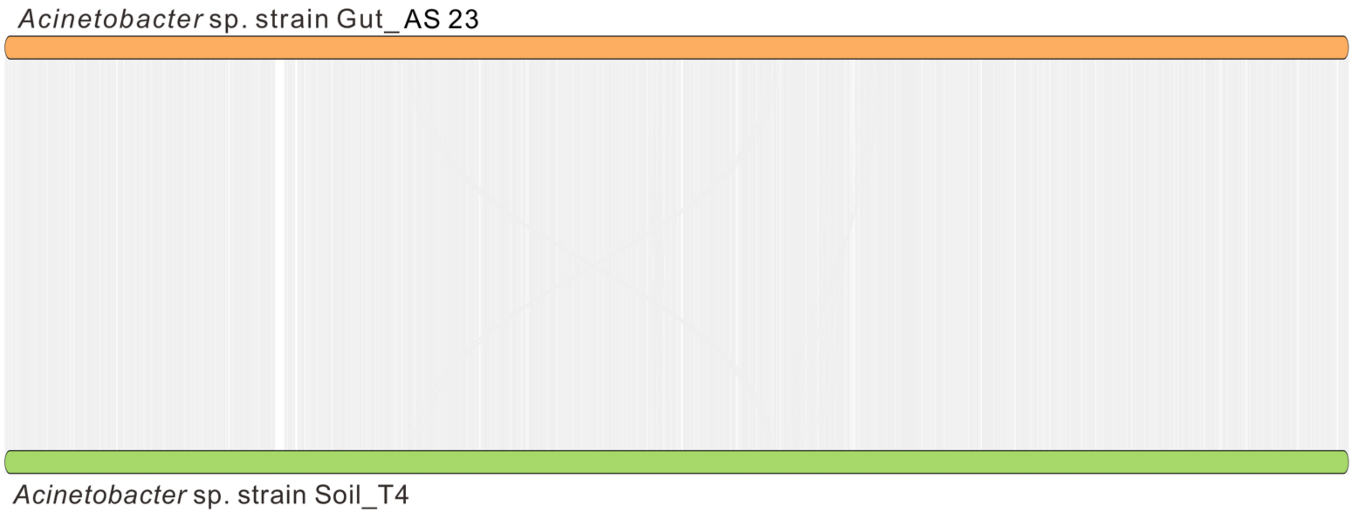

Fig. S4 Hierarchical clustering analysis of communities from gut (a) and soil (b) microbiomes (based on Bray-Curtis distances) reared on different clone plants. Panel on the left is a hierarchical clustering dendrogram indicating sample similarities. Shorter branch lengths between samples indicate higher similarity between samples. The panel on the right shows a stacked histogram of the 10 most abundant genera.

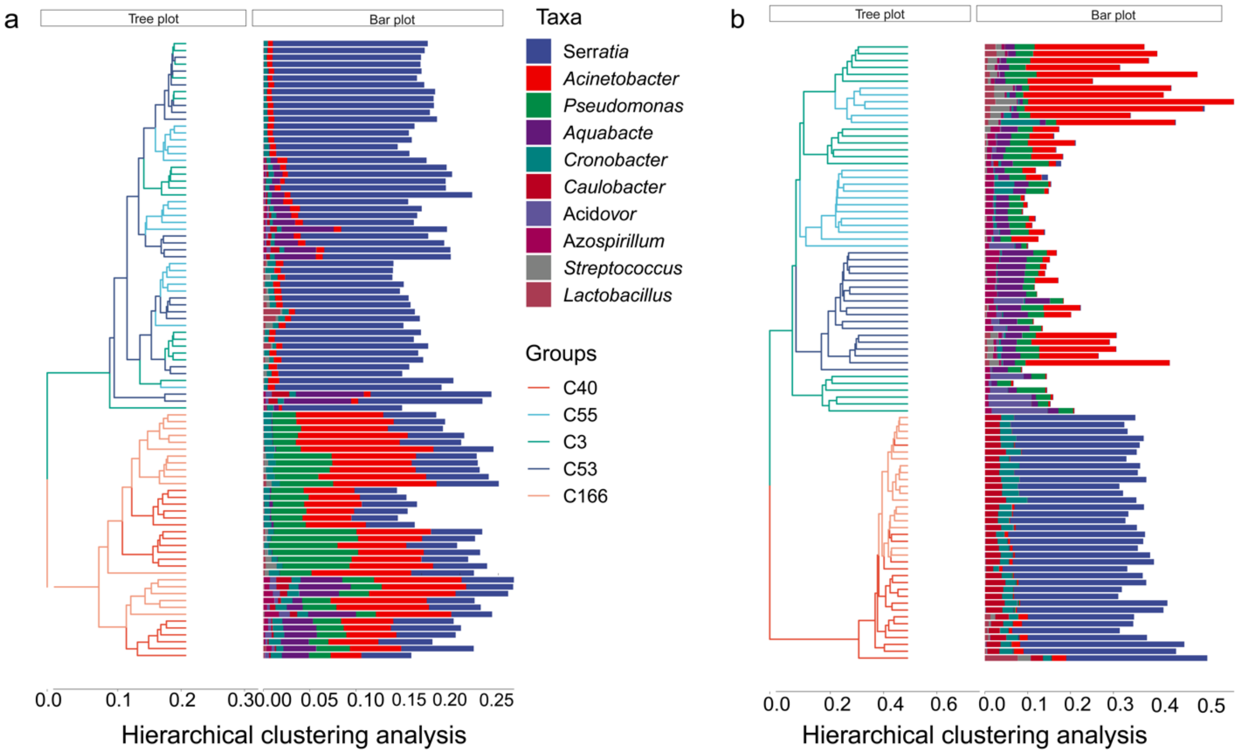

Fig. S5 Analysis of enrichment difference of ASVs level.

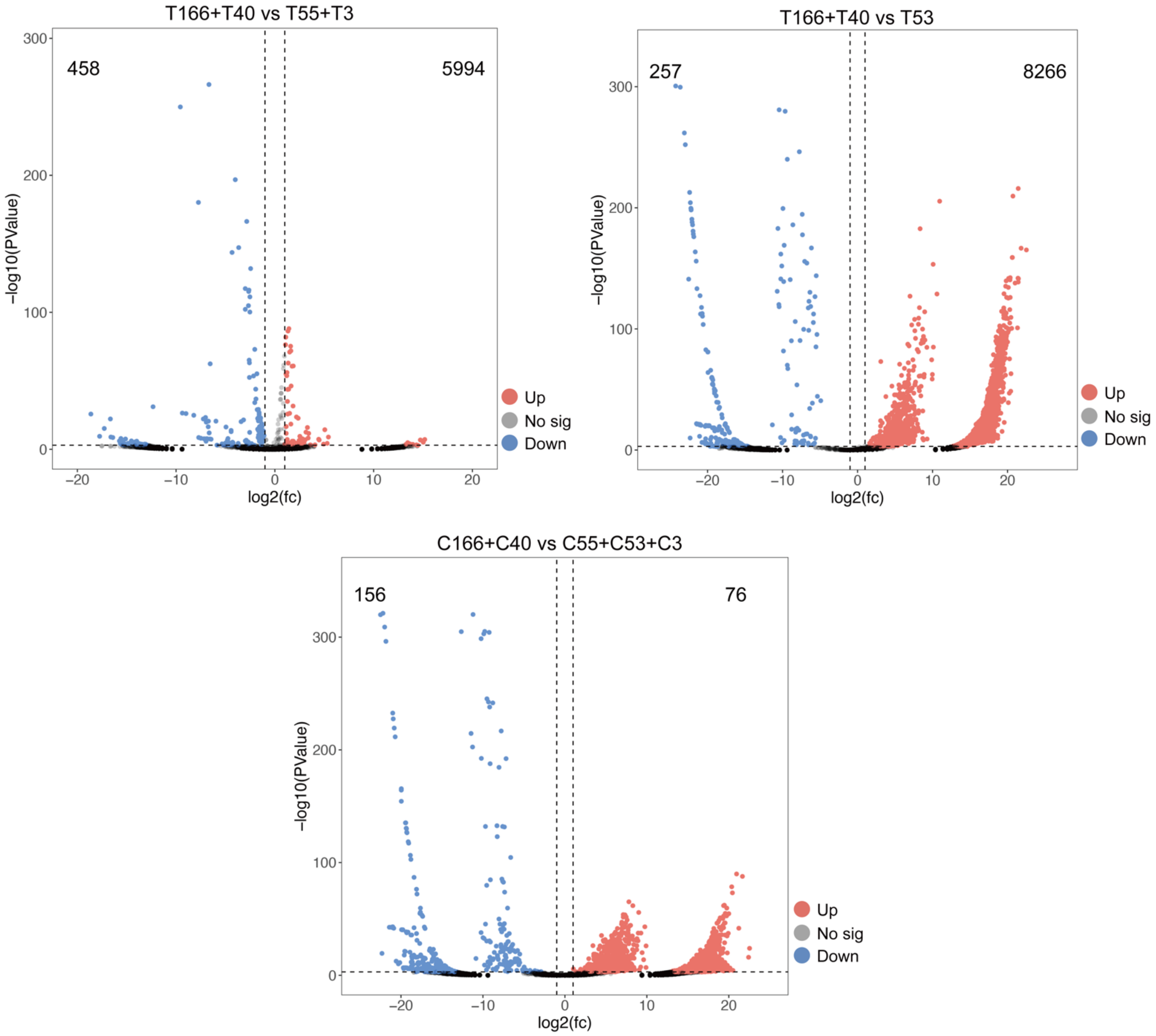

Fig. S6 Correlation between relative abundance of genus level flora and content of tea saponin.

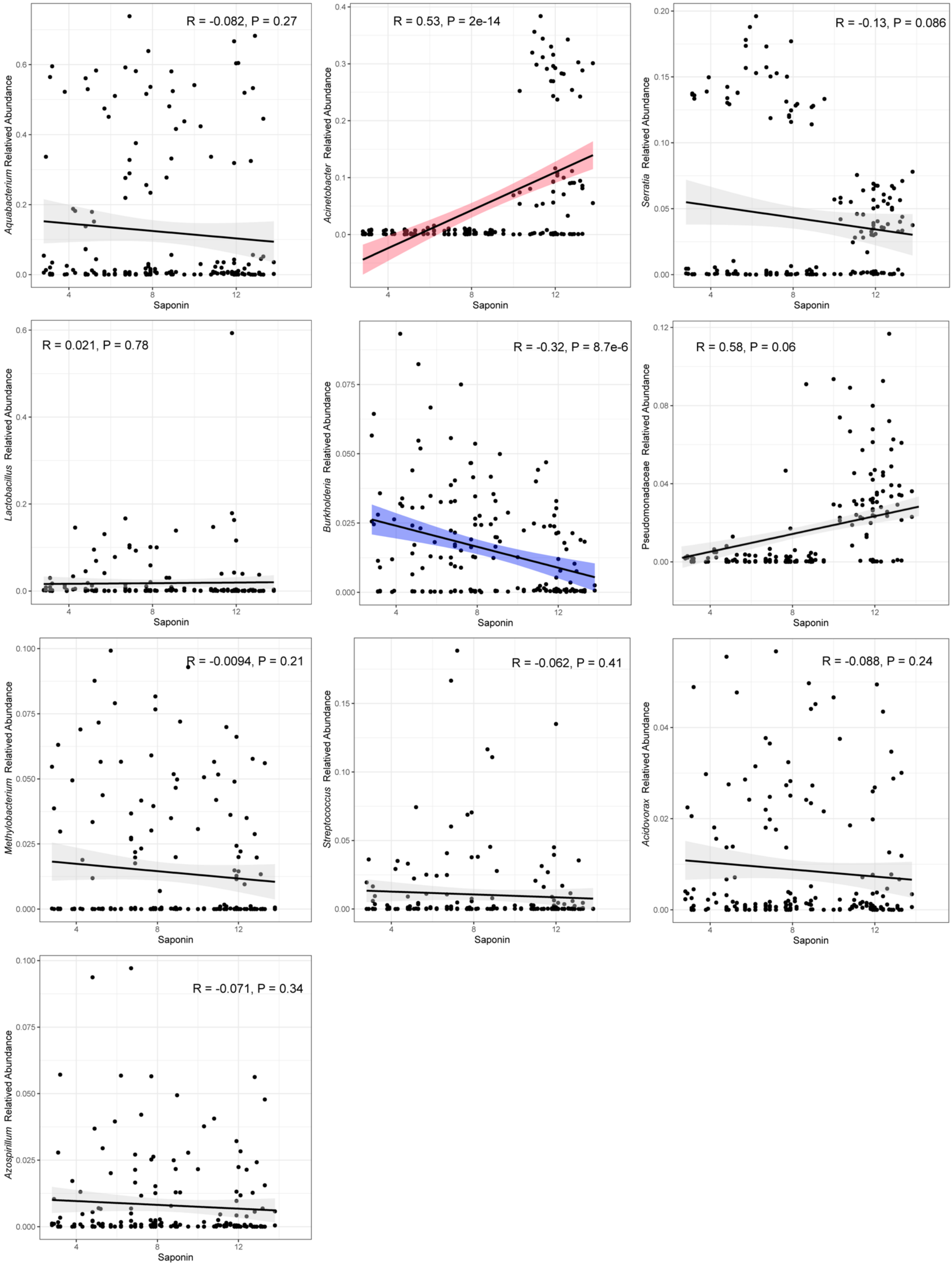

Fig. S7 Functional difference analysis of microbiome during tea saponin degradation.

TE, TM, and TL refer to tea saponin treated soil for 24h, 48h and 72h respectively. GE, GM, and GL substituted tea saponin feed for larvae for 24h, 48h and 72h, respectively. **a.** Functional difference analysis of soli microbiome during tea saponin degradation. b. Functional difference analysis of gut microbiome during tea saponin degradation.

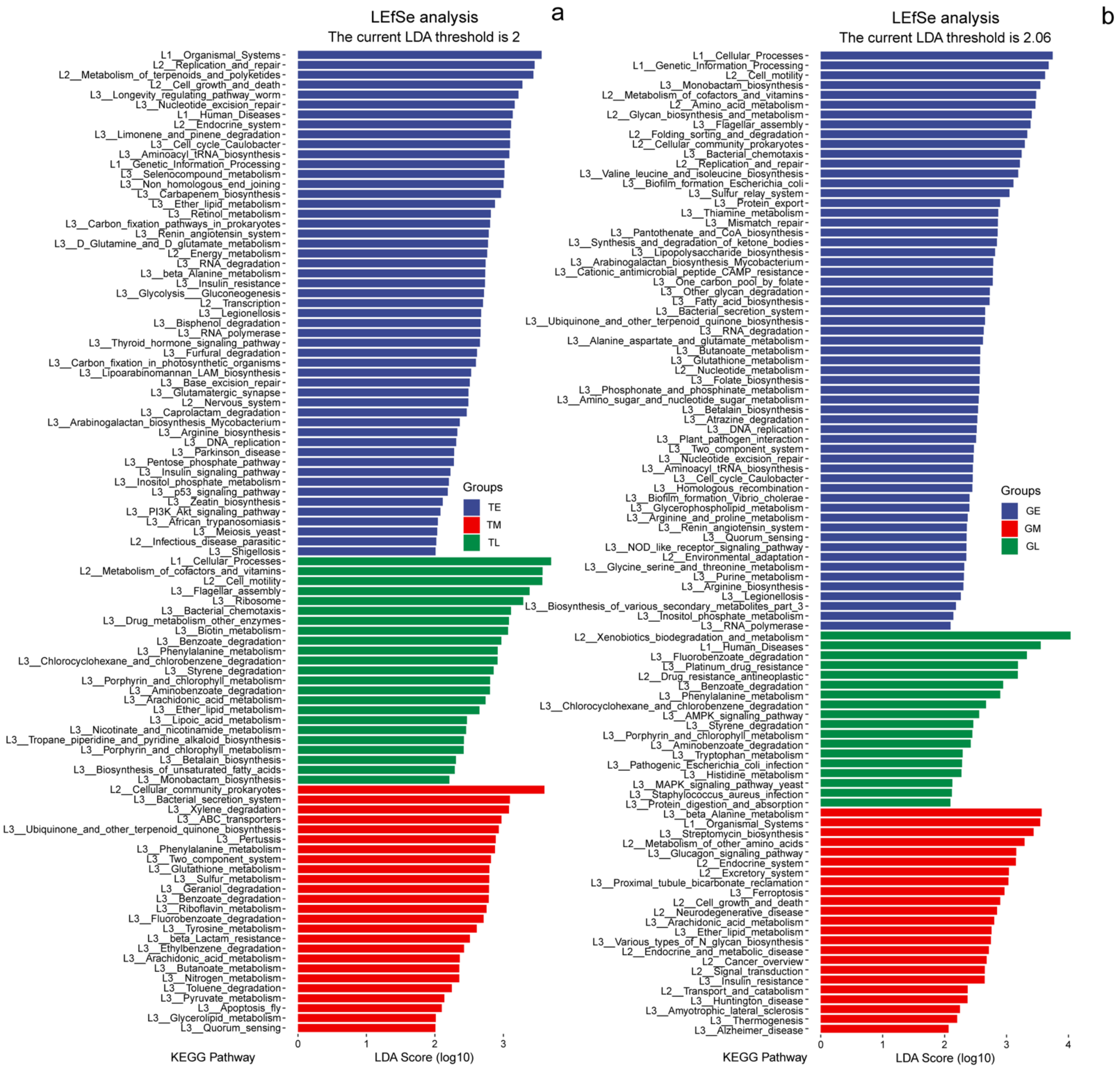

Fig. S8 Functional KEGG pathway network of *Acinetobacter* genomes during degradation of tea saponin.

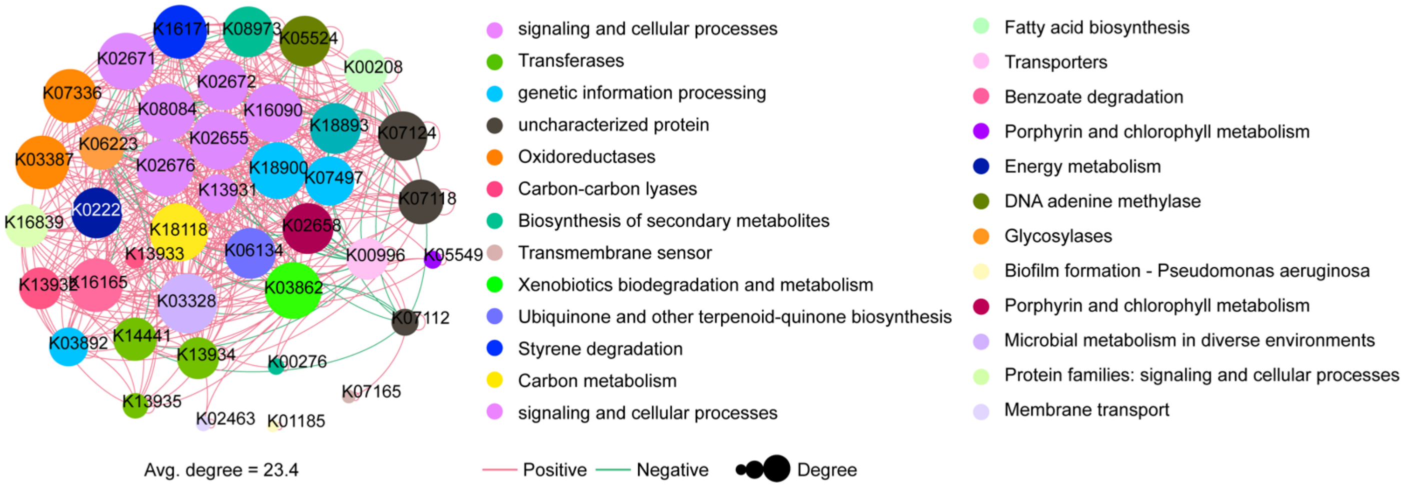

Fig. S9 Green fluorescence imaging of larva guts of the collected larvae and the fluorescence imaging of the gut. **a, c, and e,** Green fluorescence imaging of AS23 (*Acinetobacter* sp. Strain_23) in the guts of the collected larvae. **b, d, and f,** AS 23 (*Acinetobacter* sp. Strain_23) GFP luminescence after intestinal tissue autofluorescence was excluded. **g**, i, and **k**, Green fluorescence imaging of control group larva gut. **h**, **j**, and **l**, The control group' gut excluded intestinal tissue fluorescence.

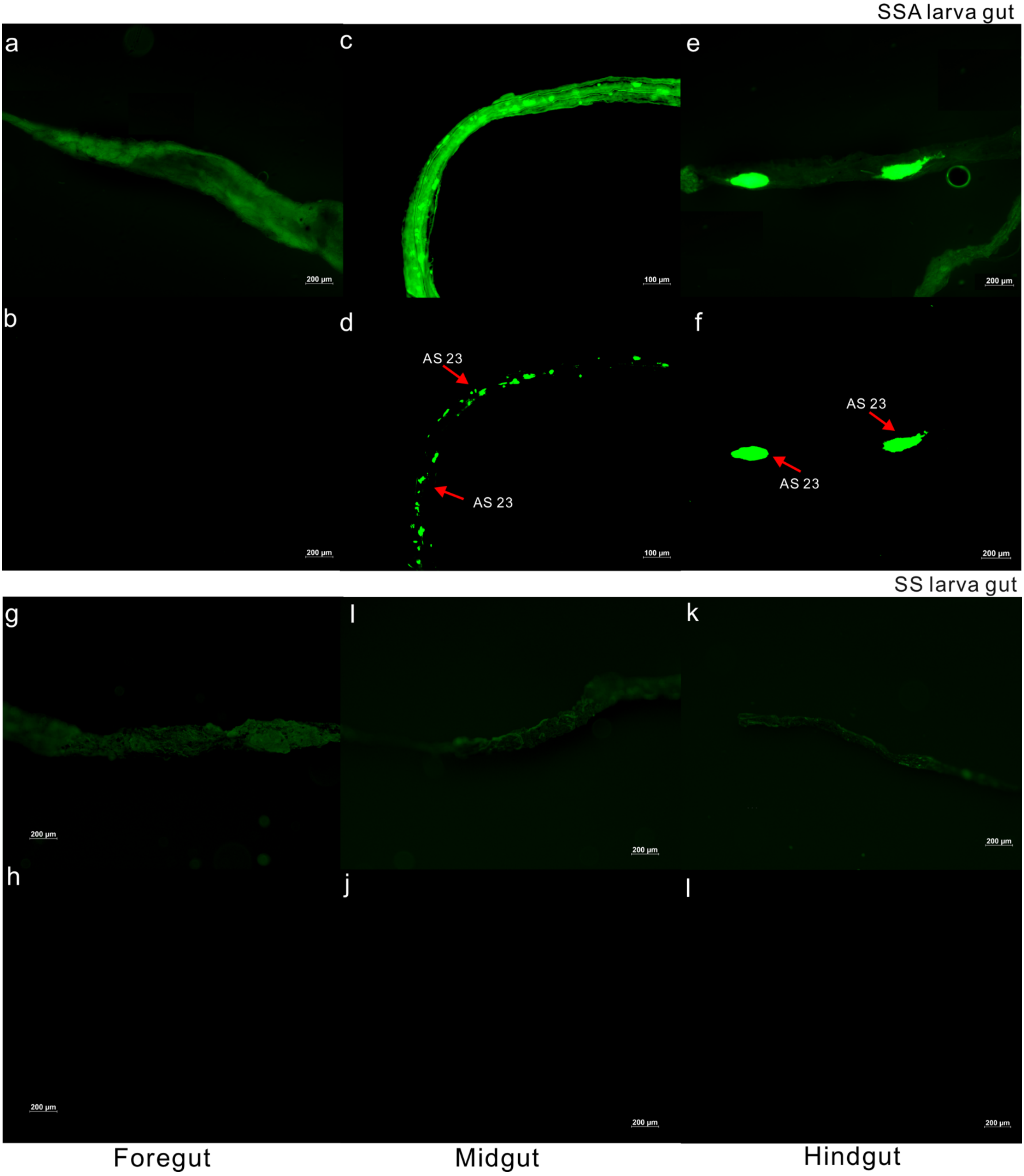

Supplement: Supplementary file 2 — Additional file 1: Figure S1. Species composition and alpha index of the microbiota of samples from soil, fruit, and gut. a, Phylum- and genus-level distributions of microbial communities recovered from fruit, soil, and weevil gut microbiota. The relative abundances of taxa that could not be annotated to the genus level are excluded from these plots. b. alpha index values for microbial communities from soil, fruits, and weevil guts. The horizontal bars within boxes represent medians. The tops and bottoms of boxes represent the 75th and 25th percentiles, respectively. The upper and lower whiskers extend to data no more than 1.5× the interquartile range from the upper edge and lower edge of the box, respectively. T: Soil, G: Fruit, C: Gut. Figure S2. Unconstrained PCoA with bray–curtis distance showing that three sources of the microbiome occur separately from each other (p = 0.001, PERMANOVA test and Anosim test). a. All samples were differentiated according to different sources. b. Unconstrained PCoA with Bray-curtis distance showing the clustering of soil samples. c. Unconstrained PCoA with Bray-curtis distance showing the clustering of fruit samples. d. Unconstrained PCoA with Braycurtis distance showing the clustering of gut samples. Figure S3. Collinearity of the Acinetobacter sp. genomes from soil and gut. The Step MCScanX software package from TBtools was used to analyze the collinearity of the two genomes. Figure S4. Hierarchical clustering analysis of communities from gut (a) and soil (b) microbiomes (based on Bray-Curtis distances) reared on different clone plants. Panel on the left is a hierarchical clustering dendrogram indicating sample similarities. Shorter branch lengths between samples indicate higher similarity between samples. The panel on the right shows a stacked histogram of the 10 most abundant genera. Figure S5. Analysis of enrichment difference of ASVs level. Figure S6. Correlation between relative abundance of genus level flora and content of [file 40168_2022_1290_MOESM1_ESM.pdf]
