# Supplementary material for: Soil-derived bacteria endow Camellia weevil with more ability to resist plant chemical defense
Source: Microbiome. 2022 Jun 25;10:97. doi: 10.1186/s40168-022-01290-3 (PMC9233397; doi:10.1186/s40168-022-01290-3)
Supplement: Supplementary file 3 — Additional file 2: Table S1. Multivariate analysis of variance based on bray–curtis distance among all samples. Table S2. Nonparametric multivariate analysis of variance between all samples based on bray–curtis distance. Table S3. Multivariate analysis of variance based on bray–curtis distance among soil and gut samples. Table S4. Nonparametric multivariate analysis of variance based on bray–curtis distance. Table S5. KEGG information annotated by Acinetobacter in Binning analysis [file 40168_2022_1290_MOESM2_ESM.pdf]

Soil-derived bacteria endow *Camellia* weevil with more ability to resist plant chemical defense

Shou-ke Zhang[1,2], Zi-kun Li[2], Jin-ping Shu[3,*], Huai-jun Xue[4], Kai Guo[2], Xu-dong Zhou[1,2,*]

[1]State Key Laboratory of Subtropical Silviculture, Zhejiang A&F University, Hangzhou, Zhejiang 311300, P. R. China.

[2]School of Forestry and Biotechnology, Zhejiang A&F University, Hangzhou, Zhejiang 311300, P. R. China.

[3]Research Institute of Subtropical Forestry, Chinese Academy of Forestry, Hangzhou, Zhejiang 311400, P. R. China.

[4]College of Life Sciences, Nankai University, Tianjin 300071, P. R. China.

*Correspondence to: Xu-dong Zhou, School of Forestry and Biotechnology, Zhejiang A&F University, Hangzhou, China. E-mail: xudong.zhou@zafu.edu.cn

Table S1. Multivariate analysis of variance based on bray–curtis distance among all samples.

Group1	Group2	Samplesize	Permutations	Anosim		PERMANOVA	
				R	pvalue	pseudoF	pvalue
all	-	270	999	0.569	0.001	34.421	0.001
QTT	QZT	60	999	0.063	0.036	3.207	0.016
QTT	JDT	60	999	0.122	0.004	4.600	0.003
QTT	QTG	60	999	0.584	0.001	28.930	0.001
QTT	QZG	60	999	0.383	0.001	15.101	0.001
QTT	JDG	60	999	0.658	0.001	24.556	0.001
QTT	QTC	60	999	0.486	0.001	31.436	0.001
QTT	QZC	60	999	0.729	0.001	36.967	0.001
QTT	JDC	60	999	0.735	0.001	37.435	0.001
QZT	JDT	60	999	0.054	0.050	1.628	0.163
QZT	QTG	60	999	0.662	0.001	44.095	0.001
QZT	QZG	60	999	0.391	0.001	14.456	0.001
QZT	JDG	60	999	0.643	0.001	21.248	0.001
QZT	QTC	60	999	0.452	0.001	27.030	0.001
QZT	QZC	60	999	0.625	0.001	30.783	0.001
QZT	JDC	60	999	0.636	0.001	31.043	0.001
JDT	QTG	60	999	0.672	0.001	46.108	0.001
JDT	QZG	60	999	0.378	0.001	16.787	0.001
JDT	JDG	60	999	0.465	0.001	15.287	0.001
JDT	QTC	60	999	0.530	0.001	27.113	0.001
JDT	QZC	60	999	0.628	0.001	28.089	0.001
JDT	JDC	60	999	0.521	0.001	27.630	0.001
QTG	QZG	60	999	0.813	0.001	45.386	0.001
QTG	JDG	60	999	0.980	0.001	56.002	0.001
QTG	QTC	60	999	1.000	0.001	266.319	0.001
QTG	QZC	60	999	1.000	0.001	304.060	0.001
QTG	JDC	60	999	1.000	0.001	325.837	0.001
QZG	JDG	60	999	0.814	0.001	20.042	0.001
QZG	QTC	60	999	0.884	0.001	56.773	0.001
QZG	QZC	60	999	0.917	0.001	65.140	0.001
QZG	JDC	60	999	0.905	0.001	65.885	0.001
JDG	QTC	60	999	1.000	0.001	72.286	0.001
JDG	QZC	60	999	1.000	0.001	74.795	0.001
JDG	JDC	60	999	1.000	0.001	74.321	0.001
QTC	QZC	60	999	0.186	0.001	7.544	0.004
QTC	JDC	60	999	0.356	0.001	20.143	0.001
QZC	JDC	60	999	0.206	0.002	8.101	0.003

Table S2. Nonparametric multivariate analysis of variance between all samples based on bray–curtis distance.

#	Df	SumsOfSqs	MeanSqs	F.Model	R^2	Pr(>F)
Groups	8	47.445009	5.930626	34.420606	0.513391	0.001
Residuals	261	44.969964	0.172299	NaN	0.486609	NaN
Total	269	92.414973	NaN	NaN	1	NaN

Table S3. Multivariate analysis of variance based on bray–curtis distance among soil and gut samples.

Group1	Group2	Samplesize	Permutations	Anosim		PERMANOVA	
				R	pvalue	pseudoF	pvalue
all	-	90	999	0.804	0.001	37.011	0.001
T40	T55	36	999	1.000	0.001	53.435	0.001
T40	T3	36	999	0.999	0.001	48.524	0.001
T40	T53	36	999	1.000	0.001	77.974	0.001
T40	T166	36	999	0.352	0.001	7.915	0.001
T55	T3	36	999	0.516	0.001	6.721	0.001
T55	T53	36	999	0.830	0.001	13.964	0.001
T55	T166	36	999	1.000	0.001	60.490	0.001
T3	T53	36	999	0.793	0.001	12.269	0.001
T3	T166	36	999	1.000	0.001	54.746	0.001
T53	T166	36	999	1.000	0.001	89.520	0.001
all	-	90	999	0.635	0.001	49.295	0.001
C40	C55	36	999	0.935	0.001	56.249	0.001
C40	C3	36	999	0.992	0.001	90.175	0.001
C40	C53	36	999	0.977	0.001	69.304	0.001
C40	C166	36	999	0.194	0.002	5.750	0.003
C55	C3	36	999	0.329	0.001	12.728	0.001
C55	C53	36	999	0.112	0.033	4.357	0.010
C55	C166	36	999	0.955	0.001	63.620	0.001
C3	C53	36	999	0.079	0.045	3.583	0.027
C3	C166	36	999	0.991	0.001	90.846	0.001
C53	C166	36	999	0.984	0.001	69.008	0.001

Table S4. Nonparametric multivariate analysis of variance based on bray–curtis distance.

#	Df	SumsOfSqs	MeanSqs	F.Model	R^2	Pr(>F)
Soil groups	4	18.890329	4.722582	37.010639	0.63526	0.001
Residuals	85	10.846057	0.127601	NaN	0.36474	NaN
Total	89	29.736386	NaN	NaN	1	NaN
Gut groups	4	3.250509	0.812627	49.294562	0.698772	0.001
Residuals	85	1.401236	0.016485	NaN	0.301228	NaN
Total	89	4.651744	NaN	NaN	1	NaN

Table S5. KEGG information annotated by *Acinetobacter* in Binning analysis

KO	Pathway	Description
K05549	Benzoate degradation	benA-xylX; benzoate/toluate 1,2-dioxygenase subunit alpha [EC:1.14.12.10 1.14.12.-]
K02658	Biofilm formation - Pseudomonas aeruginosa	pilH; twitching motility two-component system response regulator PilH
K00276	Biosynthesis of secondary metabolites	AOC3, AOC2, tynA; primary-amine oxidase
K08973		K08973; putative membrane protein
K18118	Carbon metabolism	aarC, cat1; succinyl-CoA:acetate CoA-transferase [EC:2.8.3.18]
K13932	Carbon-carbon lyases	mdcD; malonate decarboxylase beta subunit [EC:4.1.1.87]
K13933		mdcE; malonate decarboxylase gamma subunit [EC:4.1.1.87]
K06223	DNA adenine methylase	dam; DNA adenine methylase [EC:2.1.1.72]
K05524	Energy metabolism	fdxA; ferredoxin
K00208	Fatty acid biosynthesis	fabI; enoyl-[acyl-carrier protein] reductase
K03892	Genetic information processing	arsR; ArsR family transcriptional regulator, arsenate/arsenite/antimonite-responsive transcriptional repressor
K07497		K07497; putative transposase
K18900		bpeT; LysR family transcriptional regulator, regulator for bpeEF and oprC
K01185	Glycosylases	E3.2.1.17; lysozyme [EC:3.2.1.17]
K18893	Membrane transport	vcaM; ATP-binding cassette, subfamily B, multidrug efflux pump
K16839	Microbial metabolism in diverse environments	hpxO; FAD-dependent urate hydroxylase [EC:1.14.13.113]
K03387	Oxidoreductases	ahpF; alkyl hydroperoxide reductase subunit F [EC:1.6.4.-]
K07336		K07336; PKHD-type hydroxylase [EC:1.14.11.-]
K03328	polysaccharide transporter	TC.PST; polysaccharide transporter, PST family
K02226	Porphyrin and chlorophyll metabolism	cobC, phpB; alpha-ribazole phosphatase [EC:3.1.3.73]
K02463	Protein families: signaling and cellular processes	gspN; general secretion pathway protein N
K02655	Signaling and cellular processes	PIK3R1_2_3; phosphoinositide-3-kinase regulatory subunit alpha/beta/delta
K02671		PRKCA; classical protein kinase C alpha type [EC:2.7.11.13]
K02672		pilW; type IV pilus assembly protein PilW
K02676		pilZ; type IV pilus assembly protein PilZ

K08084		fimT; type IV fimbrial biogenesis protein FimT
K13931		mdcC; malonate decarboxylase delta subunit
K16090		fiu; catecholate siderophore receptor
K16171	Styrene degradation	faaH; fumarylacetoacetate (FAA) hydrolase [EC:3.7.1.2]
K13934		mdcG; phosphoribosyl-dephospho-CoA transferase [EC:2.7.7.66]
K13935	Transferases	mdcH; malonate decarboxylase epsilon subunit [EC:2.3.1.39]
K14441		rimO; ribosomal protein S12 methylthiotransferase [EC:2.8.4.4]
K07165	Transmembrane sensor	fecR; transmembrane sensor
K16165	Transporters	nagK; fumarylpyruvate hydrolase [EC:3.7.1.20]
K06134	Ubiquinone and other terpenoid-quinone biosynthesis	COQ7; 3-demethoxyubiquinol 3-hydroxylase [EC:1.14.99.60]
K07112		K07112; uncharacterized protein
K07118	Uncharacterized protein	K07113; uncharacterized protein
K07124		K07114; uncharacterized protein
K00996	Undecaprenyl-phosphate galactose phosphotransferase	rfbP; undecaprenyl-phosphate galactose phosphotransferase [EC:2.7.8.6]
K03862	Xenobiotics biodegradation and metabolism	vanA; vanillate monooxygenase [EC:1.14.13.82]
